# Socioeconomic and regional inequalities in health-related quality of life in Bhutan: a nationally representative cross-sectional study

**DOI:** 10.7189/jogh.16.04151

**Published:** 2026-05-29

**Authors:** Md Mizanur Rahman, Ying Yao, Pempa Pempa, Yot Teerawattananon, Thomas Rouyard, Ryota Nakamura

**Affiliations:** 1 Hitotsubashi Institute for Advanced Study, Hitotsubashi University, Tokyo, Japan; 2 Faculty of Economics, Keio University, Japan; 3 Health Intervention and Technology Assessment Division, Department of Health Service, Ministry of Health, Bhutan; 4 Health Intervention and Technology Assessment Program, Ministry of Public Health, Thailand; 5 Saw Swee Hock School of Public Health, National University of Singapore, Singapore; 6 Graduate School of Public Health and Health Policy, City University of New York, New York, New York, USA; 7 Department of Global Health and Development, London School of Hygiene and Tropical Medicine, London, UK

## Abstract

**Background:**

Bhutan lacks comprehensive data on health-related quality of life (HRQoL) despite its growing population and rising disease burden. This study provides the first national and subnational assessment of HRQoL inequalities and their determinants in Bhutan’s general population.

**Methods:**

We analysed data from 11 340 adults aged ≥15 years from the 2023 Bhutan National Health Survey, a nationally representative cross-sectional survey. We assessed health status using the EuroQol 5-Dimension 5 Level (EQ-5D-5L) descriptive system and the EuroQol Visual Analogue Scale (EQ-VAS). We used regression analyses to examine factors associated with HRQoL and individual EQ-5D-5L dimensions, as well as concentration, slope, and relative indices of inequality to assess national and subnational socioeconomic disparities.

**Results:**

Pain/discomfort was the most commonly reported EQ-5D-5L problem (38%; 95% confidence interval (CI) = 36, 40), followed by mobility limitations and anxiety/depression (both 14%; 95% CI = 13, 15). The mean EQ-5D-5L score was 0.95 (standard deviation (SD) = 0.10), and the mean EQ-VAS score was 77.06 (SD = 17.44). HRQoL was lower among women, older respondents, those with lower education, unemployed individuals, rural residents, and those with poorer dietary habits. In regression analyses, older age and rural residence were consistently associated with higher odds of reported problems, while higher education was protective across all EQ-5D-5L dimensions. Higher household expenditure was generally associated with higher EQ-5D and EQ-VAS scores, but the richest quintile also had higher odds of reporting problems in several individual dimensions. Inequality analyses showed larger disparities by education and area of residence than by wealth, with broadly similar patterns across gender groups and across EQ-5D and EQ-VAS scores.

**Conclusions:**

Significant socioeconomic, geographic, and behavioural disparities in HRQoL exist in Bhutan, with inequalities more pronounced by education and area of residence than by wealth. Targeted interventions for less-educated and rural populations, alongside efforts to promote healthier diets, are needed to help mitigate these inequities.

Evaluating and monitoring population health and well-being are essential for effective health policy planning and development. Health-related quality of life (HRQoL), a multidimensional measure of perceived health and well-being, provides valuable data for these efforts. As a core component in calculating quality-adjusted life years (QALYs), HRQoL enables economic evaluations of health interventions within local contexts, supporting informed, context-specific policy decisions. QALYs have become instrumental in guiding resource allocation and efforts to reduce health inequalities [1]. Optimising the distribution of health resources is especially relevant in low- and middle-income countries (LMICs) such as Bhutan, where health care budgets are constrained, and substantial sociodemographic disparities in health outcomes persist [2,3].

In Bhutan, health and well-being are closely tied to the country’s development philosophy of gross national happiness (GNH), which explicitly links public policy to promoting happiness and well-being. Article nine of the Constitution mandates that the state create conditions enabling the pursuit of GNH, and its four pillars – sustainable and equitable economic development, environmental conservation, cultural preservation, and good governance – integrate health and well-being into national planning [4]. This policy orientation underpins Bhutan’s approach to universal health coverage (UHC); the country operates a tax-funded health system that provides free access to essential modern and traditional health services for its 0.76 million citizens [5,6].

Despite this strong policy commitment, structural and geographic barriers continue to impede equitable access to health care. Bhutan’s mountainous terrain creates significant obstacles to access to services, particularly for rural communities. Although most of the population (96.8%) lives within two hours of a health centre [7], rural residents continue to use substantially fewer health care services than urban residents [8]. Service availability is further constrained by workforce shortages. In 2024, Bhutan had 24.8 medical doctors, nurses and midwives per 10 000 population [7], well below the 44.5 per 10 000 benchmark used by the World Health Organization (WHO) to assess workforce availability for progress toward UHC [9].

Compounding these structural challenges is Bhutan’s shifting epidemiological profile. Noncommunicable diseases (NCDs), including cardiovascular diseases, cancer, diabetes, and chronic respiratory disease, now account for a large and growing share of the country’s disease burden, representing 71.6% of deaths in 2021 [10]. This transition is closely linked to behavioural risk factors, including unhealthy diet, tobacco use, harmful alcohol consumption, and physical inactivity [11,12]. For example, alcohol consumption is a relevant concern in Bhutan, where per capita consumption exceeds the regional average (4.4 *vs*. 3.6 L of pure alcohol per year among people aged ≥15 years in 2022) [13]; globally, alcohol consumption contributes to more than 200 disease and injury conditions and was responsible for an estimated 2.6 million deaths in 2019 [14]. These modifiable risk factors are major contributors to NCD-related morbidity and premature mortality [15] and are also associated with lower HRQoL [16,17]. Yet evidence remains limited regarding how HRQoL varies across population groups in many LMICs, particularly with respect to sociodemographic and behavioural factors.

The evidence base in South Asia illustrates this limitation. As of 31 July 2024, our systematic literature search identified 35 relevant studies, but these were marked by three key gaps: uneven geographic coverage, ranging from ten studies in Thailand to none in Bhutan; methodological limitations, including small samples sizes and facility-based study designs; and a narrow focus on specific conditions or population subgroups rather than the general population (Table S1 in the **Online Supplementary Document**). The absence of Bhutan-specific evidence is particularly concerning because findings from other countries may not adequately capture Bhutan’s unique health system context and constraints.

To address this gap, we conducted the first nationally representative assessment of HRQoL in Bhutan. We measured HRQoL and examined inequalities and their determinants across demographic, socioeconomic, geographic, behavioural, and physiological factors to inform equitable health system planning and NCD prevention.

## METHODS

### Data

We used data from the 2023 Bhutan National Health Survey (NHS), conducted by the Ministry of Health in collaboration with the National Statistics Bureau of Bhutan. The NHS was a nationally representative, cross-sectional household survey that employed a stratified, multi-stage sampling design to ensure coverage across all 20 districts and both urban and rural strata. Sampling was based on probability proportional to district population size, with enumeration areas serving as primary sampling units (PSUs). In remote rural areas, smaller administrative units called ‘chiwogs’ were used as PSUs. Households within selected PSUs were randomly selected using circular systematic sampling.

A total of 40 617 individuals from 11 340 households were interviewed. Our analysis focused on 11 340 adults aged ≥15 years who completed the EuroQol 5-Dimension 5 Level (EQ-5D-5L) and EuroQol Visual Analogue Scale (EQ-VAS) modules; individuals with missing responses on key variables were excluded. Missing data were rare (<5%) and handled using complete-case analysis; sensitivity analyses confirmed the robustness of the results.

Data were collected through face-to-face interviews by trained enumerators using standardised questionnaires. The interviews covered four main areas: demographic and socioeconomic characteristics, medical history, health-related habits (*e.g.* diet, smoking, alcohol use) and other respondent attributes. The term ‘beliefs’ was removed from the survey scope because these questions were not analysed. To help participants with varying literacy levels accurately assess their HRQoL, interviewers used graphical illustrations to explain EQ-5D-5L severity levels. Enumerators received rigorous training and adhered to strict quality-control procedures in accordance with the latest EQ-VT protocol to ensure data consistency and accuracy [18].

### Health status assessment

Participants’ health status was measured using the EQ-5D-5L and EQ-VAS, a widely used international tool developed by the EuroQol group consisting of two standardised scales. The EQ-5D-5L descriptive system evaluates five health-related dimensions: mobility, self-care, usual activities, pain/discomfort, and anxiety/depression, with each rated across five levels of severity (no problems, slight problems, moderate problems, severe problems, and extreme problems/unable to perform). The EQ-VAS is a self-rated scale in which participants rate their overall health from zero (worst imaginable health) to 100 (best imaginable health) (Table S2 in the **Online Supplementary Document**).

### HRQoL utility score calculation

Enumerators administered the English version of the EQ-5D-5L and EQ-VAS questionnaires, translating the questions into local languages and dialects during data collection. To calculate HRQoL utility scores (EQ scores), we applied the Indian EQ-5D-5L value set, as a Bhutanese-specific value set is not currently available. The Indian value set was selected for its geographic proximity and prior use in South Asian contexts.

EQ scores summarise HRQoL by assigning weights to responses across the five dimensions of the EQ-5D-5L, based on the preferences of the reference population (in this case, the Indian population). Scores range from less than zero (worse than death) to one (perfect health), with zero representing death. To assess the robustness of the results to the choice of value set, we conducted sensitivity analyses using a crosswalk EQ-5D-3L value set derived from the Thai valuation. Thailand was selected because it is frequently used as a regional comparator in health utility analyses.

### Factors considered in HRQoL analysis

We examined factors influencing HRQoL at the individual, household, and community levels. Individual-level factors included demographic characteristics (age, sex, education, marital status, and employment status), health-related habits (fruit and vegetable intake, smoking, and alcohol use), and biometric measures (body mass index (BMI) and blood pressure). Education was categorised as no formal education, primary (grades one to six), secondary (grades seven to 12), or tertiary (college or university). Employment status was grouped as employed (government, private sector, or self-employed), unemployed, or not in the labour force (students, homemakers, or retired).

BMI was calculated as weight (kg) divided by height squared (m^2^) and classified as underweight (<18.5 kg/m^2^), normal (18.5–24.9 kg/m^2^), overweight (25.0–29.9 kg/m^2^), or obese (>30 kg/m^2^), following WHO guidelines [19]. Blood pressure was categorised as poor (systolic ≥140 mmHg or diastolic ≥90 mmHg), intermediate (systolic 120–139 mmHg or diastolic 80–89 mmHg, or controlled to <120/<80 mmHg with treatment), or ideal (systolic <120 mmHg or diastolic <80 mmHg without medication). Dietary habits were assessed using weekly fruit and vegetable consumption, classified as poor (zero to one day per week), intermediate (two to three days per week), or ideal (≥4 days per week).

Household-level factors included household size and per-capita consumption expenditure. Household expenditure quintiles were derived by ranking households by per-capita consumption and dividing them into five equal groups from poorest to richest. Community-level factors included area of residence (urban or rural) and geographic region. The 20 Bhutanese districts were grouped into Eastern, Central, and Western regions, as defined by the country’s administrative classifications.

### Statistical analysis

We performed descriptive analyses for the entire sample and across demographic subgroups (*e.g.* age, sex, employment status), calculating percentages, means, and standard deviations to summarise data and identify patterns in the three HRQoL measures. Sex differences in mean HRQoL outcomes were examined using independent two-sample *t* tests. Differences across age, place of residence, region, marital status, employment status, educational level, and expenditure quintile were examined using one-way analysis of variance.

The distribution of health states often exhibits skewness and multimodality, with many individuals reporting perfect health (score of one) and gaps between perfect and less-than-perfect states. Conventional linear regression models, such as ordinary least squares, may not accurately capture this variability. To address these characteristics, we employed three tailored regression models to investigate the influence of the considered factors: Tobit regression for EQ-5D scores to account for censoring at perfect health; generalised linear regression for EQ-VAS scores; and binary multilevel logistic regression for each EQ-5D dimension, categorising responses as ‘no problems’ (zero) or ‘any level of problems’ (one).

To evaluate socioeconomic disparities in HRQoL, we used the slope index of inequality (SII) and the relative index of inequality (RII). The SII measures the absolute difference in HRQoL between the most and least advantaged individuals, while the RII expresses this difference relative to the population average. These indices were estimated using regression methods: binary logit regression for each EQ-5D-5L dimension, and linear regression for the EQ-5D-5L index and EQ-VAS scores. An RII>1 indicates that HRQoL is concentrated among richer groups, whereas an RII<1suggests concentration among disadvantaged groups. In addition, we used the concentration index, which quantifies socioeconomic-related inequality as twice the area between the concentration curve and the line of equality. The concentration index ranges from −1 to +1, with negative values indicating that the health variable is concentrated among poorer groups and positive values indicating that it is concentrated among richer groups. All statistical analyses were conducted using Stata, version 17 (StataCorp, College Station, Texas, USA), and *R*, version 4.3.2 (R Core Team, Vienna, Austria), and statistical significance was defined as a two-sided *P*-value <0.05.

## RESULTS

### Study population

The sample was 62.2% female, with an average height of 157.33 cm and a weight of 65.08 kg. Mean blood pressure was 125.52/84.21 mmHg, and ages ranged from 15–19 years (5.1%) to >60 years (11.6%). About half had completed secondary education, with employment at 74.0% for men and 43.0% for women. The majority (74.4%) were married (**Table 1**).

**Table 1:** Characteristics of the study population*

Variables	Both sexes (n = 11 340)	Male (n = 4289)	Female (n = 7051)	*P*-value†
Age in years, x̄	40.89	41.77	40.34	<0.001
Height in cm, x̄	157.33	163.46	153.34	<0.001
Weight in kg, x̄	65.08	69.34	62.36	<0.001
SBP in mmHg, x̄	125.52	127.86	124.05	<0.01
DBP in mmHg, x̄	84.21	84.90	83.78	>0.05
Age in years				<0.001
*15–19*	579 (5.1)	243 (5.7)	336 (4.8)	
*20–29*	1989 (17.5)	715 (16.7)	1274 (18.1)	
*30–39*	3191 (28.1)	1071 (25.0)	2120 (30.1)	
*40–49*	2510 (22.1)	918 (21.4)	1592 (22.6)	
*50–59*	1751 (15.4)	749 (17.5)	1002 (14.2)	
*60–69*	1236 (10.9)	549 (12.8)	687 (9.7)	
*≥70*	84 (0.7)	44 (1.0)	40 (0.6)	
Education				<0.001
*No formal education*	4595 (40.5)	1507 (35.1)	3088 (43.8)	
*Primary or less*	1632 (14.4)	644 (15.0)	988 (14.0)	
*Secondary or equivalent*	4231 (37.3)	1655 (38.6)	2576 (36.5)	
*Tertiary*	881 (7.8)	483 (11.3)	398 (5.7)	
Marital status				<0.001
*Never married*	1642 (14.5)	790 (18.4)	852 (12.1)	
*Married*	8442 (74.4)	3187 (74.3)	5255 (74.5)	
*Other (divorced/widowed/separated)*	1256 (11.1)	312 (7.3)	944 (13.4)	
Working status				<0.001
*Working*	6231 (55.0)	3175 (74.1)	3056 (43.3)	
*Not working*	5107 (45.0)	1112 (25.9)	3995 (56.7)	
BMI				<0.001
*Underweight*	402 (3.6)	171 (4.0)	231 (3.3)	
*Normal weight*	4695 (42.0)	2070 (48.8)	2625 (37.8)	
*Overweight*	4040 (36.1)	1515 (35.7)	2525 (36.3)	
*Obese*	2054 (18.4)	485 (11.4)	1569 (22.6)	
Blood pressure				>0.1
*Poor*	1796 (15.9)	667 (15.6)	1129 (16.1)	
*Intermediate*	5989 (53.1)	2264 (53.0)	3725 (53.1)	
*Ideal*	3498 (31.0)	1342 (31.4)	2156 (30.8)	
Fruits consumption				>0.1
*Poor*	2819 (24.9)	1086 (25.3)	1733 (24.6)	
*Intermediate*	5127 (45.2)	1909 (44.5)	3218 (45.6)	
*Ideal*	3394 (29.9)	1294 (30.2)	2100 (29.8)	
Vegetable consumption				>0.1
*Poor*	108 (1.0)	43 (1.0)	65 (0.9)	
*Intermediate*	536 (4.7)	198 (4.6)	338 (4.8)	
*Ideal*	10 696 (94.3)	4048 (94.4)	6648 (94.3)	
Place of residence				>0.1
*Urban*	4217 (37.2)	1564 (36.5)	2653 (37.6)	
*Rural*	7123 (62.8)	2725 (63.5)	4398 (62.4)	
Region of residence				<0.01
*East*	2980 (26.3)	1089 (25.4)	1891 (26.8)	
*West*	5250 (46.3)	2071 (48.3)	3179 (45.1)	
*Central*	3110 (27.4)	1129 (26.3)	1981 (28.1)	

BMI – body mass index, DBP – diastolic blood pressure, SBP – systolic blood pressure, x̄ – mean

*Presented as n (%) unless specified otherwise.

†*P*-values were derived using *t* tests for continuous variables and χ^2^ test for categorical variables.

### Health status

**Figure 1:**
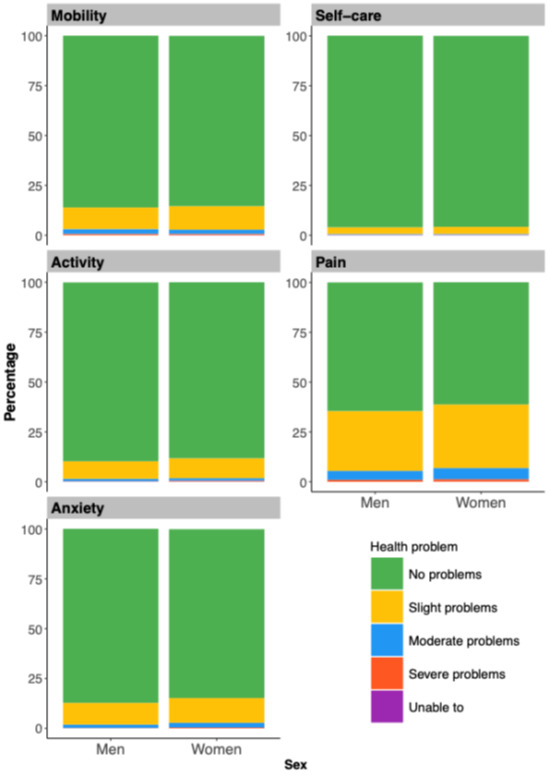
Distribution of reported health problems across EQ-5D-5L dimensions, by gender. EQ-5D – EuroQol 5-Dimension.

Our results showed uneven distributions across all EQ-5D-5L dimensions, with many respondents reporting ‘no problems’ (**Figure 1**; Table S3 in the **Online Supplementary Document**). Pain/discomfort was the most commonly reported issue, affecting 37.5% of participants, followed by mobility and anxiety/depression (both 14.3%). Problems with usual activities and self-care were less prevalent, reported by 11.3% and 4.1%, respectively.

Gender differences were evident across various dimensions, with women reporting higher rates of mobility problems (14.7% *vs*. 13.9%) and significantly more pain/discomfort and anxiety/depression than men. No notable gender differences were observed in self-care or usual activities. Age-related trends revealed a sharp increase in mobility issues after age 70, while self-care, usual activities, and pain/discomfort problems rose progressively with age for both genders. Men experienced a more pronounced age-related increase in limitations of usual activities. Notably, anxiety/depression peaked in the 15–19 age group, highlighting distinct mental health challenges for this cohort (Tables S4–6 in the **Online Supplementary Document**).

### HRQoL utility scores

**Figure 2:**
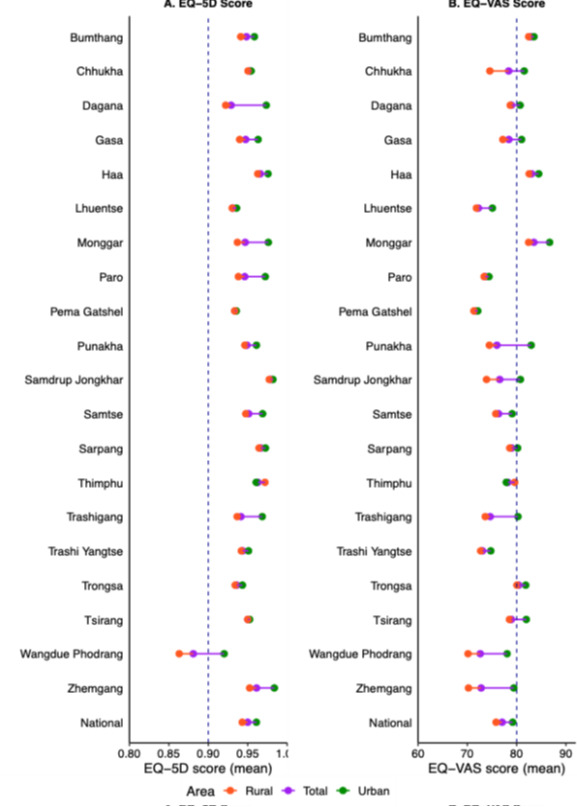
National and subnational level EQ-5D and EQ-VAS scores by area of residence. **Panel A.** EQ-5D score. **Panel B.** EQ-Vas score. EQ-VAS – EuroQol Visual Analogue Scale, EQ-5D – EuroQol 5-Dimension.

Descriptive analyses revealed variations in HRQoL utility scores across demographic and socioeconomic groups (Table S7 in the **Online Supplementary Document**). Men had higher scores than women, with HRQoL peaking in the 20–29 age group and declining steadily to its lowest in those aged ≥70. Higher education and employment were associated with better HRQoL, while wealth did not follow the expected trend; the richest reported slightly lower scores than middle-income groups. Additionally, urban residents had higher HRQoL scores than rural residents at both national and district levels (**Figure 2**, Panel A and B). At the national level, the mean EQ-5D index was 0.950 using the Indian value set and 0.875 using the Thai value set, yielding a mean difference of −0.074 (95% confidence interval (CI) = −0.075, −0.074, *P* < 0.001); across districts, absolute EQ-5D levels varied markedly, but the direction and magnitude of differences between value sets were consistent (Table S8 in the **Online Supplementary Document**).

### Determinants of HRQoL

#### EQ-5D dimension

Age was strongly associated with reported problems across all EQ-5D dimensions, with older respondents generally more likely to experience difficulties. Men had lower odds of reporting pain/discomfort (odds ratio (OR) = 0.84; 95% CI = 0.76, 0.93) and anxiety/depression (OR = 0.79; 95% CI = 0.69, 0.91) than women. Education was protective across all dimensions, with tertiary education associated with substantially lower odds of problems compared with no formal education for mobility limitations (OR = 0.34; 95% CI = 0.23, 0.49), for self-care difficulties (OR = 0.35; 95% CI = 0.17, 0.73), and for problems with usual activities (OR = 0.37; 95% CI = 0.25, 0.55) (**Table 2**).

**Table 2:** Binary multilevel logistic regression analyses on reported health problems, OR (95% CI)

Variables	Mobility	Self-care	Activity	Pain/discomfort	Anxiety/depression
Age in years					
*15–19*	Ref.	Ref.	Ref.	Ref.	Ref.
*20–29*	0.92 (0.63, 1.35)	1.23 (0.63, 2.40)	1.22 (0.81, 1.83)	0.68 (0.53, 0.87)	0.82 (0.60, 1.14)
*30–39*	0.97 (0.64, 1.46)	1.51 (0.73, 3.10)	1.40 (0.90, 2.17)	0.68 (0.52, 0.89)	0.84 (0.59, 1.20)
*40–49*	1.54 (1.01, 2.35)	1.99 (0.93, 4.26)	1.59 (1.00, 2.51)	0.92 (0.69, 1.22)	0.87 (0.60, 1.26)
*50–59*	2.31 (1.50, 3.55)	3.55 (1.65, 7.62)	1.94 (1.22, 3.11)	1.13 (0.84, 1.51)	0.80 (0.54, 1.18)
*60–69*	3.90 (2.52, 6.05)	5.78 (2.68, 12.48)	3.29 (2.05, 5.28)	1.47 (1.08, 1.98)	0.82 (0.54, 1.22)
*≥70*	5.81 (3.02, 11.20)	11.50 (4.04, 32.73)	3.80 (1.84, 7.85)	2.14 (1.21, 3.76)	0.74 (0.35, 1.55)
Gender					
*Female*	Ref.	Ref.	Ref.	Ref.	Ref.
*Male*	1.00 (0.87, 1.14)	1.14 (0.90, 1.46)	0.94 (0.81, 1.09)	0.84 (0.76, 0.93)	0.79 (0.69, 0.91)
Education					
*No formal education*	Ref.	Ref.	Ref.	Ref.	Ref.
*Primary or below*	1.02 (0.86, 1.21)	0.97 (0.71, 1.34)	0.83 (0.68, 1.01)	0.96 (0.84, 1.10)	0.96 (0.80, 1.15)
*Secondary or equivalent*	0.74 (0.62, 0.89)	0.96 (0.69, 1.32)	0.75 (0.62, 0.91)	0.77 (0.68, 0.87)	0.78 (0.66, 0.93)
*Tertiary*	0.34 (0.23, 0.49)	0.35 (0.17, 0.73)	0.37 (0.25, 0.55)	0.69 (0.57, 0.84)	0.78 (0.60, 1.02)
Marital status					
*Never married*	Ref.	Ref.	Ref.	Ref.	Ref.
*Married*	0.69 (0.54, 0.88)	0.74 (0.48, 1.15)	0.72 (0.56, 0.94)	0.86 (0.73, 1.02)	0.75 (0.61, 0.94)
Other*	0.88 (0.66, 1.17)	0.90 (0.54, 1.50)	1.04 (0.76, 1.41)	0.98 (0.80, 1.20)	1.41 (1.09, 1.83)
Working status					
*Working*	Ref.	Ref.	Ref.	Ref.	Ref.
*Not working*	1.19 (1.04, 1.36)	1.97 (1.56, 2.49)	1.32 (1.14, 1.52)	1.04 (0.95, 1.15)	1.10 (0.96, 1.25)
Fruit intake					
*Poor*	Ref.	Ref.	Ref.	Ref.	Ref.
*Intermediate*	0.81 (0.70, 0.94)	0.87 (0.67, 1.13)	1.02 (0.87, 1.20)	0.83 (0.74, 0.92)	0.88 (0.76, 1.01)
*Ideal*	0.79 (0.67, 0.93)	0.85 (0.64, 1.14)	1.01 (0.84, 1.21)	0.88 (0.78, 0.99)	0.93 (0.79, 1.09)
Vegetable intake					
*Poor*	Ref.	Ref.	Ref.	Ref.	Ref.
*Intermediate*	0.89 (0.47, 1.69)	0.78 (0.24, 2.50)	0.39 (0.21, 0.74)	0.57 (0.37, 0.89)	0.90 (0.48, 1.72)
*Ideal*	1.09 (0.61, 1.95)	1.05 (0.37, 2.98)	0.63 (0.36, 1.10)	0.68 (0.45, 1.01)	1.13 (0.63, 2.02)
BMI					
*Underweight*	Ref.	Ref.	Ref.	Ref.	Ref.
*Normal weight*	0.96 (0.68, 1.37)	1.02 (0.55, 1.87)	1.12 (0.76, 1.65)	1.51 (1.17, 1.94)	1.27 (0.91, 1.77)
*Overweight*	1.10 (0.77, 1.57)	0.98 (0.53, 1.81)	1.02 (0.69, 1.51)	1.56 (1.21, 2.02)	1.07 (0.76, 1.50)
*Obese*	1.27 (0.87, 1.84)	0.77 (0.40, 1.48)	0.90 (0.59, 1.36)	1.51 (1.15, 1.99)	0.95 (0.66, 1.37)
Blood pressure					
*Poor*	Ref.	Ref.	Ref.	Ref.	Ref.
*Intermediate*	0.94 (0.80, 1.10)	1.00 (0.75, 1.33)	1.07 (0.89, 1.28)	1.00 (0.89, 1.13)	1.14 (0.96, 1.35)
*Ideal*	0.99 (0.82, 1.20)	1.17 (0.83, 1.64)	1.13 (0.92, 1.40)	1.09 (0.94, 1.25)	1.09 (0.90, 1.33)
Expenditure quintile					
*Quintile 1 (poorest)*	Ref.	Ref.	Ref.	Ref.	Ref.
*Quintile 2*	0.88 (0.73, 1.06)	0.77 (0.55, 1.07)	0.87 (0.70, 1.06)	1.07 (0.94, 1.23)	1.22 (1.01, 1.47)
*Quintile 3*	1.01 (0.83, 1.22)	0.95 (0.68, 1.31)	0.93 (0.76, 1.15)	1.09 (0.95, 1.26)	1.07 (0.88, 1.30)
*Quintile 4*	0.99 (0.81, 1.20)	0.82 (0.58, 1.16)	0.88 (0.70, 1.09)	1.27 (1.10, 1.46)	1.26 (1.03, 1.53)
*Quintile 5 (richest)*	1.34 (1.11, 1.62)	1.03 (0.74, 1.44)	1.34 (1.09, 1.65)	1.52 (1.32, 1.76)	1.41 (1.16, 1.72)
Area of residence					
*Urban*	Ref.	Ref.	Ref.	Ref.	Ref.
*Rural*	1.41 (1.17, 1.69)	1.32 (0.94, 1.86)	1.62 (1.32, 2.00)	1.22 (1.06, 1.40)	1.22 (1.01, 1.47)

BMI – body mass index, Ci – confidence interval, OR – odds ratio, ref – reference

*Others refers to divorced/widowed/separated; model adjusted for household size, self-reported diabetes, and region of residence.

Significant differences were also observed by household expenditure and area of residence. Unexpectedly, compared with the poorest quintile, the richest quintile had higher odds of reporting problems with mobility (OR = 1.34; 95% CI = 1.11, 1.62), usual activities (OR = 1.34; 95% CI = 1.09, 1.65), pain/discomfort (OR = 1.51; 95% CI = 1.30, 1.74), and anxiety/depression (OR = 1.41; 95% CI = 1.16, 1.72). Rural residents consistently reported more problems than urban residents, with ORs of 1.41 for mobility (95% CI = 1.11, 1.69), 1.62 for usual activities (95% CI = 1.32, 2.00), 1.22 for pain/discomfort (95% CI = 1.06, 1.40), and 1.22 for anxiety/depression (95% CI = 1.01, 1.47).

Dietary habits were also significantly associated with HRQoL. Compared with poor fruit intake, ideal fruit intake was associated with lower odds of pain/discomfort (OR = 0.77; 95% CI = 0.66, 0.91). Similarly, compared with poor vegetable intake, intermediate vegetable intake was associated with substantially lower odds of problems with usual activities (OR = 0.39; 95% CI = 0.21, 0.74) and pain/discomfort (OR = 0.57; 95% CI = 0.37, 0.89).

Biometric measures were mainly associated with pain/discomfort. Relative to underweight individuals, the odds of reporting pain/discomfort were higher among those with normal weight (OR = 1.51; 95% CI = 1.17–1.94), overweight (OR = 1.56; 95% CI = 1.21–2.02), and obesity (OR = 1.51; 95% CI = 1.15–1.99).

#### EQ-5D and EQ-VAS scores

Higher HRQoL was generally observed among younger respondents, those with higher education, employed and married individuals, urban residents, and those reporting healthier dietary habits (**Table 3**).

**Table 3:** Regression analyses on health-related quality of life scores

Predictors	EQ-5D score (Tobit regression)		EQ-VAS score (OLS regression)	
	**Estimates (95% CI)**	***P*-value**	**Estimates (95% CI)**	***P*-value**
Age in years				
*15–19*	0		0	0
*20–29*	0.00 (–0.01, 0.01)	0.84	–1.01 (–2.78, 0.77)	0.27
*30–39*	–0.01 (–0.02, 0.01)	0.31	–2.22 (–4.15, –0.28)	0.02
*40–49*	–0.01 (–0.03, 0.00)	0.01	–3.78 (–5.82, –1.75)	<0.01
*50–59*	–0.02 (–0.04, –0.01)	<0.01	–5.63 (–7.74, –3.52)	<0.01
*60–69*	–0.05 (–0.06, –0.03)	<0.01	–7.11 (–9.30, –4.92)	<0.01
*≥70*	–0.07 (–0.10, –0.05)	<0.01	–10.47 (–14.64, –6.30)	<0.01
Gender				
*Female*	0		0	0
*Male*	0.01 0.00, 0.01)	0.01	0.62 (–0.10, 1.33)	0.09
Education				
*No formal education*	0		0	
*Primary or less*	0.01 (0.00, 0.01)	0.04	0.52 (–0.47, 1.51)	0.30
*Secondary or equivalent*	0.01 (0.00, 0.01)	<0.01	1.45 (0.54, 2.37)	<0.01
*Tertiary*	0.02 (0.01, 0.03)	<0.01	3.39	<0.01
Marital status				
*Never married*	0		0	0
*Married*	0.01 (0.01, 0.02)	<0.01	1.86 (0.68, 3.05)	<0.01
Other*	0.00 (–0.01, 0.01)	0.82	0.02 (–1.48, 1.52)	0.98
Working status				
*Working*	0		0	0
*Not working*	–0.01 (–0.01, 0.00)	<0.01	–1.79 (–2.49, –1.10)	<0.01
BMI				
*Underweight*	0		0	0
*Normal weight*	–0.01 (–0.02, 0.00)	0.16	0.16 (–1.63, 1.95)	0.86
*Overweight*	–0.01 (–0.02, 0.00)	0.26	1.18 (–0.65, 3.01)	0.21
*Obese*	0.00 (–0.02, 0.01)	0.45	0.64 (–1.30, 2.57)	0.52
Blood pressure				
*Poor*	0		0	0
*Intermediate*	0.00 (–0.01, 0.00)	0.17	0.12 (–0.79, 1.04)	0.79
*Ideal*	–0.01 (–0.01, 0.00)	0.05	–0.64 (–1.69, 0.41)	0.23
Fruit intake				
*Poor*	0		0	0
*Intermediate*	0.01 (0.00, 0.01)	<0.01	1.06 (0.27, 1.85)	0.01
*Ideal*	0.01 (0.00, 0.01)	<0.01	1.51 (0.64, 2.39)	<0.01
Fruit/vegetable intake				
*Poor*	0		0	
*Intermediate*	0.02 (0.00, 0.04)	0.12	0.75 (–2.78, 4.27)	0.68
*Ideal*	0.01 (–0.01, 0.03)	0.37	1.78 (–1.45, 5.01)	0.28
Expenditure quintile				
*Quintile 1 (poorest)*	0		0	
*Quintile 2*	0.00 (–0.01, 0.00)	0.29	–0.93 (–1.92, 0.07)	0.07
*Quintile 3*	–0.01 (–0.01, 0.00)	0.05	–1.56	<0.01
*Quintile 4*	–0.01 (–0.01, 0.00)	0.02	–2.05 (–3.07, –1.04)	<0.01
*Quintile 5 (richest)*	–0.02 (–0.02, –0.01)	<0.01	–3.94 (–4.97, –2.92)	<0.01
Area of residence				
*Urban*	0		0	
*Rural*	–0.01 (–0.01, –0.01)	<0.01	–2.54 (–3.27, –1.82)	<0.01
R^2^ Nagelkerke	–0.010		0.043	

BMI – body mass index, CI – confidence interval, EQ-VAS – EuroQol Visual Analogue Scale, EQ-5D – EuroQol 5-Dimension

*Self-reported diabetes, household size and region of residence were adjusted in this model.

Associations with household expenditure differed somewhat from those observed in the binary logistic regression analyses of EQ-5D dimensions. HRQoL scores increased monotonically across household expenditure quintiles, with participants in the fourth and fifth quintiles reporting significantly higher scores than those in the poorest quintile. These results were robust to alternative EQ-5D value sets, with similar patterns observed using both Indian and Thai tariffs (Table S9 in the **Online Supplementary Document**).

#### Magnitude of inequalities in HRQoL

**Figure 3:**
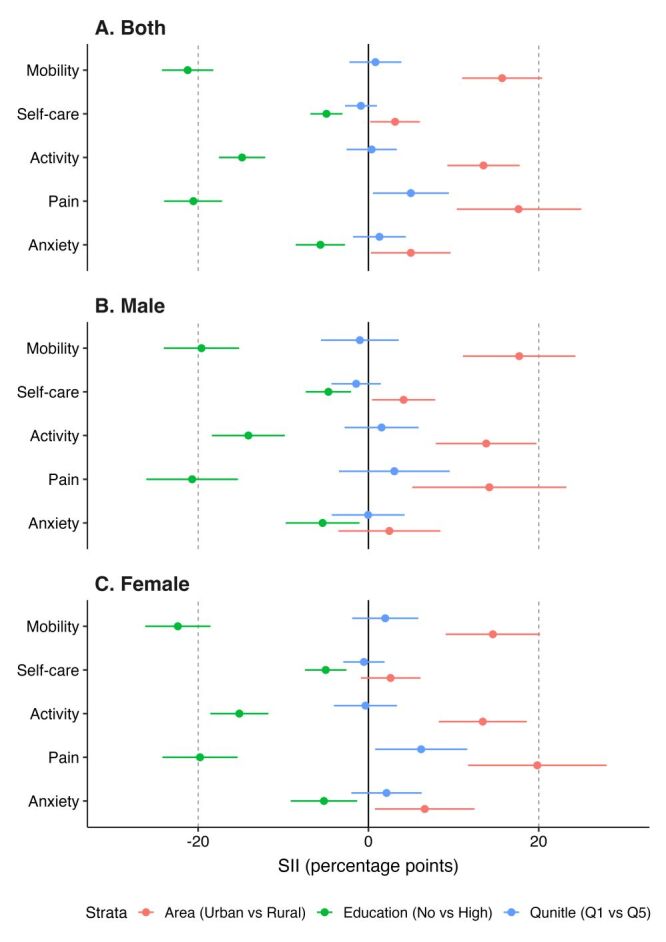
Socioeconomic disparities in EQ-5D-5L health domains, by gender. **Panel A.** Both. **Panel B.** Male. **Panel C.** Female. EQ-5D – EuroQol 5-Dimension.

The SII results indicate significant disparities across EQ-5D-5L health dimensions by education, household expenditure quintile, and area of residence, with broadly similar patterns across gender groups (**Figure 3**, Panel A–C; Table S10 in the **Online Supplementary Document**).

Compared with younger individuals, older individuals were more likely to report pain/discomfort (25 percentage points (pp)), mobility issues (24 pp), usual activity limitations (14 pp), and self-care difficulties (7 pp). Wealth-related disparities showed a mixed pattern: although higher household expenditure was generally associated with fewer reported problems, wealthier individuals reported a slightly higher prevalence of pain/discomfort (5 pp) than poorer groups. Education showed a consistent gradient across all dimensions, with individuals with higher education reporting significantly fewer health issues, including 21 pp fewer mobility and pain/discomfort problems, 15 pp fewer problems with usual activities, and 6 pp fewer anxiety/depression problems, compared with those with lower education.

At the national level, rural residents had utility scores 3.39 pp lower than urban residents (Figure S1 and Table S11 in the **Online Supplementary Document**). In most districts, EQ-5D scores were higher among urban populations, except in Thimphu (the capital city), where rural residents scored 2.2 pp higher. Education was consistently associated with better scores, with those holding higher education scoring 5.14 pp higher nationally (Table S12 in the **Online Supplementary Document**). At the district level, education gaps ranged from 14 pp in Wangdue Phodrang to about 2 pp in Thimphu and Samdrup Jongkhar. Wealth-based inequalities were minimal compared to disparities in residence area and education (Table S13 in the **Online Supplementary Document**). In most districts, participants in the highest expenditure quintile reported slightly lower EQ-5D than those in the lowest quintile, with the largest difference in Tsirang (6.96 pp).

Similar patterns were observed for EQ-VAS scores at both national and district levels (Figure S2 and Tables S14–16 in the **Online Supplementary Document**). RII estimates were consistent with SII results in both direction and magnitude (Figure S3 and S4 in the **Online Supplementary Document**), and concentration indices for wealth quintile and education showed similar patterns of inequality for HRQL indices at both national and subnational levels (Tables S11–16 in the **Online Supplementary Document**).

## DISCUSSION

This study is the first to investigate HRQoL and its determinants among the general population in Bhutan. Using data from a nationally representative survey, we identified significant inequalities in HRQoL across demographic and socioeconomic groups at national and district levels, with disparities linked to age, gender, education, marital status, employment, household expenditure, and residence area.

Our results showed skewed distributions across EQ-5D-5L dimensions, with a high prevalence of ‘no problems’ responses. Several factors may explain this pattern. First, it may reflect Bhutan’s generally healthier population compared to neighboring countries. Life expectancy at birth in Bhutan increased by 9.2 years, from 65.7 years in 2000 to 74.9 years in 2021, compared with a five-year rise in the broader Southeast Asian region, from 63.4 to 68.4 years [10]. Despite a growing burden of NCDs such as obesity and cardiovascular diseases, Bhutan maintains the highest ‘healthy life expectancy’ – the average years expected to live in ‘full health’ – in South Asia [10]. Second, Bhutanese cultural norms emphasising positive health perceptions may influence fewer reported health problems. Bhutanese society prioritises resilience, mindfulness, and well-being, values deeply rooted in Buddhist traditions and the nation’s GNH framework [20]. Third, the survey instrument’s limited sensitivity to minor health issues may also contribute to these findings. Similar patterns have been noted in studies from Poland [21] and Zimbabwe [22], suggesting that such skewed distributions may be a common limitation in HRQoL assessments across diverse cultural contexts.

Self-care was the least frequently reported problem (4.1%), whereas pain/discomfort was the most common (37.5%). This discrepancy may stem from cultural norms emphasising self-care and holistic well-being, as well as practical factors such as physical labour, limited access to health care, and cultural attitudes toward expressing pain [3,5]. Similar patterns have been observed globally, with pain/discomfort often among the most frequently reported problems and self-care issues generally less common [23–25]. However, other patterns have also been reported; for example, in Vietnam, problems with usual activities were most frequently reported [26].

A notable finding was the higher prevalence of anxiety and depression among participants aged 15–19. This trend may be driven by several factors related to Bhutan’s education system and societal expectations. Students in this age group face intense academic pressure and high societal expectations as they prepare for high-stakes national exams that significantly impact their educational and career prospects. Achieving top scores is critical for access to higher education and scholarships, heightening family and community pressure. Similar trends have been observed in East Asian countries like Korea and Japan, where intense competition for university admission has been linked to adverse mental health outcomes [27]. Additionally, for those earning scholarships, transitioning to study away from home often brings emotional challenges, such as loneliness and homesickness, exacerbating mental health impacts during this pivotal stage.

On average, men reported higher HRQoL scores than women, with particularly large difference in pain/discomfort and anxiety/depression. Among women, reported problems increased with age across all dimensions. Among men, problems with mobility, self-care, pain/discomfort, and anxiety/depression were less common between ages 15 and 39, but increased after age 40; problems with usual activities, however, increased more consistently with age. These trends may partly reflect gender roles and traditional socio-cultural norms in Bhutan [28], which can affect how men and women perceive, experience, and report health problems. Similar findings have been observed elsewhere. In China, reported problems increased with age across most dimensions, and women were more likely to report pain/discomfort and anxiety/depression [29,30]. Studies in other countries of the region also documented age-related increases in reported problems across HRQoL dimensions for both men and women [24,31–33].

Education and employment emerged as strong determinants of HRQoL. Respondents with secondary or tertiary education and those who were employed consistently reported higher HRQoL than individuals without formal education or those who were unemployed. These findings are consistent with evidence showing that education has a protective effect on health, with health-related behaviours (including smoking, drinking, and physical activity) and BMI mediating a substantial share of this relationship [34]. Education and employment may also support better HRQoL by improving health literacy, self-care practices, and access to health care services. Similar associations between education, employment, and perceived health or well-being have been reported in China, India, and Vietnam [23,24,26].

Our findings also revealed significant HRQoL inequalities by area of residence and wealth status at both national and district levels. Urban residents generally scored higher on the EQ-5D, which may reflect better access to health care and health-related infrastructure. While similar trends have been observed in India [24], contrasting results were reported in Vietnam, where urban residents had lower HRQoL, potentially due to higher living costs, pollution, and stress [26]. Unexpectedly, our results indicated a tendency toward pro-poor inequality in most districts and nationally, which contrasts with previous studies in the region that found a positive relationship between wealth and subjective health [35]. While this finding warrants further investigation, it might indicate that lower-income individuals in Bhutan develop greater adaptive resilience to adversity, leading them to report better subjective health despite economic challenges.

Incorporating behavioural and physiological factors into HRQoL analysis provides additional policy-relevant insight for NCD prevention. In this study, ideal fruit and vegetable intake was positively associated with HRQoL, reinforcing the value of promoting healthy diets as part of population-level prevention strategies. By contrast, higher BMI and hypertension were associated with greater reporting of pain/discomfort and mobility limitations, highlighting the importance of integrating NCD prevention, screening, and management within primary health care systems. Evidence from both high-income countries [16,17] and LMICs [36] shows that behavioural risk factors, including unhealthy diet, harmful alcohol consumption, and tobacco use, are generally associated with lower HRQoL. Although the Bhutan National Health Survey did not collect detailed tobacco and alcohol consumption data for the present analysis, future survey rounds should incorporate these behavioural risk factors to better capture their influence on HRQoL and inform targeted, equity-oriented prevention programmes.

This study provides the first comprehensive analysis of HRQoL and its determinants in Bhutan, addressing a critical evidence gap. Using a nationally representative sample of over 40 000 individuals, we offer detailed insights into HRQoL across diverse demographic and socioeconomic categories. This large sample size, rare in Bhutan, reflects the national authorities’ commitment to health monitoring and evidence-based policymaking. Additionally, using the EQ-5D-VAS tool, a widely validated measure, ensures robust assessment and international comparability. However, several limitations should be acknowledged. Reliance on self-reported data introduces risks of recall bias, social desirability bias, and under- or overreporting, potentially skewing HRQoL estimates. The survey’s cross-sectional nature limits causal inference, making it unclear whether observed associations reflect temporary fluctuations or robust trends. Longitudinal studies are needed to address this limitation and shed light on causal determinants of HRQoL. Moreover, although the sampling design and weighting accounted for population diversity and response rates, the inability to track individual responses may have led to non-response bias, potentially affecting representativeness for some subgroups. The absence of a Bhutan-specific EQ-5D value set required reliance on the Indian value set to calculate utility scores, which might not fully capture Bhutanese health preferences. Our choice of the Indian value set may not perfectly reflect Bhutanese preferences; however, sensitivity analyses using an alternative crosswalk value set produced similar rankings of HRQoL across groups, suggesting robust conclusions. The cross-sectional design and reliance on complete-case analysis mean that causality cannot be inferred and non-response bias may persist despite weighting. Future research should develop a Bhutan-specific EQ-5D value set and employ longitudinal designs.

## CONCLUSIONS

This study provides valuable and timely insights into sociodemographic disparities in HRQoL within Bhutan’s general population. Our findings highlight significant inequalities across demographic groups, emphasising the need for targeted public health interventions. Policymakers should prioritise initiatives to improve the quality of life of older adults, enhance education, and strengthen mental health services for younger populations. Additionally, efforts to increase health care access in rural areas, address behavioural risk factors such as smoking and harmful alcohol use, and support married individuals could further enhance community well-being. This study lays a foundation for informed policy decisions and paves the way for future longitudinal research to monitor evolving health trends and assess the impact of interventions over time.

## Additional material


Online Supplementary Document
