# Supplementary material for: Socioeconomic and regional inequalities in health-related quality of life in Bhutan: a nationally representative cross-sectional study
Source: J Glob Health. 2026 May 29;16:04151. doi: 10.7189/jogh.16.04151 (PMC13220650; doi:10.7189/jogh.16.04151)
Supplement: Online Supplementary Document [file jogh-16-04151-s001.pdf]

Supplement to: Rahman MM, Yao Y, Pempa P, Teerawattananon Y, Rouyard T, Nakamura R. Socioeconomic and regional inequalities in health-related quality of life in Bhutan: a nationally representative cross-sectional study. J Glob Health. 2026;16:04151.

Table S1: Study characteristics of published studies (n=35)

Study	Survey year	Country	Sample size	Focus population	Name of quality index	EQ-5D index	VAS-index
Polack et al, 2010[41]	2005-2006	Bangladesh	1212	Adults with visually impairing cataract	EQ-5D-3L	NA	NA
Sultana et al, 2016[42]	2014	Bangladesh	1433	Low-income communities	EQ-5D-3L, VAS-index	0.76	77.00
Mahumud et al, 2019[43]	2015-2016	Bangladesh	465	Pregnant women	EQ-5D-3L, VAS-index	0.67	79.00
Barua et al, 2021[44]	2018	Bangladesh	1806	Type 2 diabetes population	EQ-5D-5L	0.62	NA
Mannan et al, 2022[45]	2020-2021	Bangladesh	1912	Patients with hypertension	EQ-5D-3L	0.64, 0.59, 0.58	NA
Namdeo et al, 2023[46]	2014-2015	Bangladesh	318	Type 2 diabetes population	EQ-5D-5L, VAS-index	0.62	69.00
Singh et al, 2017[47]	2010-2011	India	16287	General population	EQ-5D-3L, VAS-index	NA	74.50
Kohler et al, 2018[48]	2014-2016	India	224	Postpartum women	EQ-5D-5L	0.77, 0.66	NA
Muhammed et al, 2018[49]	2015-2016	India	101	Patients with systemic lupus erythematosus	EQ-5D-3L, VAS-index	NA	54.30
Jyani et al, 2022[50]	2019-2020	India	2409	General population	EQ-5D-5L, VAS-index	0.85	75.18
Jyani et al, 2023[33]	2019-2020	India	2307	General population	EQ-5D-5L, VAS-index	0.84	75.28
Purba et al, 2017 (b)[51]	2015-2016	Indonesia	1054	General population	EQ-5D-5L, VAS-index	NA	79.38
Setyowibowo et al, 2018[52]	NA	Indonesia	603	Women with breast cancer symptoms	EQ-5D-5L, VAS-index	0.80	69.10
Purba et al, 2018 (a)[53]	2015	Indonesia	1754	Individuals living on Ciliwung River	EQ-5D-5L, VAS-index	0.91	81.74
Rensa et al, 2019[54]	2017	Indonesia	325	Older women with low socioeconomic status	EQ-5D-3L, VAS-index	76.80	70.00
Fitriana et al, 2022[55]	2019	Indonesia	1294	General population	EQ-5D-3L	NA	NA
Saleem et al, 2012[56]	2010	Pakistan	385	Hypertensive patients	EQ-5D-3L, VAS-index	0.46	63.97
Saleem et al, 2018[57]	2015-2016	Pakistan	226	Pulmonary tuberculosis patients who were drug-susceptible	EQ-5D-3L, VAS-index	0.43, 0.68, 0.88	54.73, 63.47, 76.56
Ahmed et al, 2021[58]	2019	Pakistan	602	HIV/AIDS and receiving antiretroviral therapy	EQ-5D-3L, VAS-index	0.39	66.20
Kularatna et al, 2014[59]	2012-2013	Sri Lanka	719	General population	EQ-5D-3L	0.85	NA
Kularatna et al, 2015[60]	2012-2013	Sri Lanka	736	General population	EQ-5D-3L	NA	NA

Damayanthi et al, 2018[61]	2015-2016	Sri Lanka	1300	Older people	EQ-5D-3L	NA	NA
Kularatna et al, 2019[62]	2015	Sri Lanka	1162	Patients with chronic kidney disease	EQ-5D-3L	0.54	NA
Senanayake et al, 2019[63]	NA	Sri Lanka	1036	Patients with chronic kidney disease	EQ-5D-3L, VAS-index	0.52	51.35
Jayamaha et al, 2023[64]	NA	Sri Lanka	464	Patients with substance use disorders	EQ-5D-5L, VAS-index	0.55, 0.81	50, 90
Sakthong et al, 2015[65]	2014-2015	Thailand	1156	Patients with various chronic diseases	EQ-5D-5L, VAS-index	0.86	78.00
Muennig et al, 2015[66]	2013	Thailand	102	Adult refugees	EQ-5D-5L	0.70	NA
Sakthong and Munpan, 2017[67]	2014-2015	Thailand	356	Patients with chronic diseases	EQ-5D-5L, VAS-index	NA	NA
Pattanaphesaj et al, 2018[68]	2013-2014	Thailand	1207	General population	EQ-5D-5L, VAS-index	NA	83.08
Janssen et al, 2019[69]	2007	Thailand	1409	General population	EQ-5D-3L, VAS-index	NA	78.90
Kangwanrattanakul and Parmontree, 2020[70]	NA	Thailand	1200	General population	EQ-5D-3L, EQ-5D-5L, VAS-index	NA	NA
Taburee et al, 2020[71]	2016	Thailand	384	Older adults	EQ-5D-3L	0.80	NA
Aung et al, 2022[72]	2019	Thailand	1509	Older adults	EQ-5D-5L, VAS-index	0.82	77.03
Kaikeaw et al, 2023[73]	2003, 2006, 2015, 2019	Thailand	12450, 12535, 29670, 16324	General population	EQ-5D-5L	0.832, 0.867, 0.934, 0.941	NA
Kangwanrattanakul and Krägeloh, 2024[74]	2023	Thailand	2000	General population	EQ-5D-3L, EQ-5D-5L, VAS-index	0.845, 0.923	79.83

Table S2: EQ-5D-5L and EQ-VAS questionnaires

Under each heading, please tick the ONE box that best describes your health TODAY.		
	MOBILITY<br>I have no problems in walking about<br>I have slight problems in walking about<br>I have moderate problems in walking about<br>I have severe problems in walking about<br>I am unable to walk about	
	SELF-CARE<br>I have no problems washing or dressing myself<br>I have slight problems washing or dressing myself<br>I have moderate problems washing or dressing myself<br>I have severe problems washing or dressing myself<br>I am unable to wash or dress myself	
	USUAL ACTIVITIES (e.g. work, study, housework, family or leisure activities)<br>I have no problems doing my usual activities<br>I have slight problems doing my usual activities<br>I have moderate problems doing my usual activities<br>I have severe problems doing my usual activities<br>I am unable to do my usual activities	
	PAIN / DISCOMFORT<br>I have no pain or discomfort<br>I have slight pain or discomfort<br>I have moderate pain or discomfort<br>I have severe pain or discomfort<br>I have extreme pain or discomfort	
	ANXIETY / DEPRESSION<br>I am not anxious or depressed<br>I am slightly anxious or depressed<br>I am moderately anxious or depressed<br>I am severely anxious or depressed<br>I am extremely anxious or depressed	
	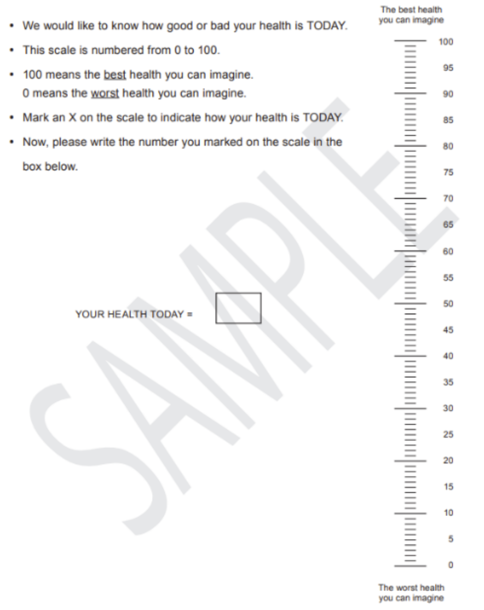<br>• We would like to know how good or bad your health is TODAY.<br>• This scale is numbered from 0 to 100.<br>• 100 means the best health you can imagine.<br>0 means the worst health you can imagine.<br>• Mark an X on the scale to indicate how your health is TODAY.<br>• Now, please write the number you marked on the scale in the box below.<br>YOUR HEALTH TODAY = 	

Table S3: Distribution of reported health problems across EQ-5D-5L dimensions, by gender

EQ-5D-5L dimensions	Frequency (%)			p-value
	Both sexes (n=11340)	Male (n=4289)	Female (n=7051)	
**Mobility**				>0.1
No problems	9709 (85.6)	3692 (86.1)	6017 (85.3)	
Slight problems	1299 (11.5)	464 (10.8)	835 (11.8)	
Moderate problems	254 (2.2)	103 (2.4)	151 (2.1)	
Severe problems	73 (0.6)	29 (0.7)	44 (0.6)	
Unable to	5 (0.0)	1 (0.0)	4 (0.1)	
**Self-care**				>0.1
No problems	10876 (95.9)	4121 (96.1)	6755 (95.8)	
Slight problems	386 (3.4)	142 (3.3)	244 (3.5)	
Moderate problems	48 (0.4)	17 (0.4)	31 (0.4)	
Severe problems	22 (0.2)	7 (0.2)	15 (0.2)	
Unable to	8 (0.1)	2 (0.1)	6 (0.1)	
**Activity**				>0.1
No problems	10062 (88.7)	3842 (89.6)	6220 (88.2)	
Slight problems	1084 (9.6)	379 (8.8)	705 (10.0)	
Moderate problems	139 (1.2)	52 (1.2)	87 (1.2)	
Severe problems	36 (0.3)	9 (0.2)	27 (0.4)	
Unable to	19 (0.2)	7 (0.2)	12 (0.2)	
**Pain/discomfort**				<0.01
No problems	7091 (62.5)	2768 (64.5)	4323 (61.3)	
Slight problems	3534 (31.2)	1287 (30.0)	2247 (31.9)	
Moderate problems	583 (5.1)	192 (4.5)	391 (5.6)	
Severe problems	126 (1.1)	41 (1.0)	85 (1.2)	
Unable to	6 (0.1)	1 (0.0)	5 (0.1)	
**Anxiety/depression**				<0.001
No problems	9712 (85.6)	3742 (87.3)	5970 (84.7)	
Slight problems	1344 (11.9)	467 (10.9)	877 (12.4)	
Moderate problems	243 (2.1)	73 (1.7)	170 (2.4)	
Severe problems	38 (0.3)	7 (0.2)	31 (0.4)	
Unable to	3 (0.0)	0 (0.0)	3 (0.0)	

Table S4: Problems in EQ-5D-5L dimensions by age groups for both sexes in Bhutan, 2023

EQ-5D-5L dimensions	Age groups (years) (%)							p-value
	15-19<br>n= 579	20-29<br>n= 1989	30-39<br>n= 3191	40-49<br>n= 2510	50-59<br>n= 1751	60-69<br>n= 1236	≥70<br>n= 84	
**Mobility (%)**								<0.001
No problems	89.9	92.8	91.6	86.0	79.1	69.0	62.0	
Slight problems	8.9	6.0	6.5	11.1	17.1	23.9	28.8	
Moderate problems	1.0	1.0	1.4	2.3	2.7	5.4	6.7	
Severe problems	0.2	0.3	0.6	0.4	1.0	1.7	2.5	
Unable to	0.0	0.0	0.0	0.1	0.1	0.1	0.0	
**Self-care (%)**								<0.001
No problems	96.8	97.9	97.5	96.7	94.1	90.3	85.2	
Slight problems	2.7	1.7	1.9	2.7	4.9	8.4	9.8	
Moderate problems	0.5	0.3	0.3	0.3	0.6	0.9	4.0	
Severe problems	0.0	0.1	0.2	0.1	0.3	0.3	1.1	
Unable to	0.0	0.0	0.1	0.2	0.1	0.1	0.0	
**Activity (%)**								<0.001
No problems	91.7	92.6	91.6	89.8	86.1	78.2	76.4	
Slight problems	7.5	6.7	7.4	8.2	11.9	18.0	14.1	
Moderate problems	0.9	0.6	0.6	1.5	1.1	3.2	5.4	
Severe problems	0.0	0.0	0.4	0.3	0.7	0.3	4.2	
Unable to	0.0	0.1	0.1	0.3	0.3	0.3	0.0	
**Pain (%)**								<0.001
No problems	64.0	70.6	68.4	61.4	55.0	49.7	37.1	
Slight problems	29.5	25.6	26.7	32.2	37.9	39.0	44.7	
Moderate problems	4.7	3.4	3.9	5.3	5.5	9.4	17.1	
Severe problems	1.7	0.4	1.0	1.1	1.5	1.9	1.1	
Unable to	0.1	0.0	0.1	0.0	0.1	0.1	0.0	
**Anxiety (%)**								>0.1
No problems	83.8	86.7	86.5	85.9	85.6	84.8	83.9	
Slight problems	13.6	11.3	11.2	11.4	11.4	12.4	11.5	
Moderate problems	2.1	1.7	1.9	2.5	2.8	2.0	4.6	
Severe problems	0.6	0.3	0.4	0.2	0.3	0.6	0.0	
Unable to	0.0	0.0	0.0	0.0	0.0	0.2	0.0	

Table S5: Problems in EQ-5D-5L dimensions by age groups for males in Bhutan, 2023

EQ-5D-5L dimensions	Age groups (years), (%)							p-value (χ^2-statistic)
	15-19 n= 243	20-29 n= 715	30-39 n= 1071	40-49 n= 918	50-59 n= 749	60-69 n= 549	≥70 n= 44	
**Mobility (%)**								<0.001
No problems	90.4	91.0	92.4	87.3	80.8	74.7	63.0	
Slight problems	8.2	7.6	5.8	9.0	15.9	19.5	28.3	
Moderate problems	1.0	1.1	1.2	3.2	2.6	4.3	4.0	
Severe problems	0.4	0.3	0.5	0.5	0.7	1.4	4.8	
Unable to	0.0	0.0	0.0	0.0	0.1	0.0	0.0	
**Self-care (%)**								<0.001
No problems	96.6	97.9	98.1	96.9	95.0	90.7	86.8	
Slight problems	3.3	1.8	1.5	2.8	4.0	8.1	8.5	
Moderate problems	0.1	0.1	0.2	0.4	0.7	0.8	2.7	
Severe problems	0.0	0.1	0.1	0.0	0.3	0.2	2.1	
Unable to	0.0	0.0	0.1	0.0	0.0	0.2	0.0	
**Activity (%)**								<0.001
No problems	92.1	91.9	93.0	90.3	87.6	83.0	73.6	
Slight problems	7.7	7.6	6.2	7.6	10.8	14.2	15.3	
Moderate problems	0.1	0.4	0.5	1.9	1.2	2.2	6.4	
Severe problems	0.0	0.0	0.1	0.2	0.4	0.2	4.8	
Unable to	0.0	0.1	0.2	0.1	0.1	0.3	0.0	
**Pain (%)**								<0.001
No problems	66.1	70.3	72.8	64.0	59.8	54.0	34.5	
Slight problems	28.1	26.3	23.3	30.7	35.0	36.0	51.8	
Moderate problems	5.5	3.3	3.3	4.6	3.9	7.8	11.6	
Severe problems	0.3	0.2	0.6	0.6	1.2	2.2	2.1	
Unable to	0.0	0.0	0.0	0.0	0.1	0.0	0.0	
**Anxiety (%)**								>0.1
No problems	82.0	88.5	88.6	88.3	87.4	86.9	81.8	
Slight problems	15.7	10.3	9.7	9.6	11.1	10.6	11.0	
Moderate problems	2.2	1.1	1.5	2.0	1.5	1.9	7.2	
Severe problems	0.0	0.1	0.2	0.1	0.0	0.6	0.0	
Unable to	0.0	0.0	0.0	0.0	0.0	0.0	0.0	

Table S6: Problems in EQ-5D-5L dimensions by age groups for females in Bhutan, 2023

EQ-5D-5L dimensions	Age groups (years) (%)							p-value (χ^2-statistic)
	15-19 n= 336	20-29 n= 1274	30-39 n= 2120	40-49 n= 1592	50-59 n= 1002	60-69 n= 687	≥70 n= 40	
**Mobility (%)**								<0.001
No problems	89.6	93.7	91.2	85.2	77.8	64.2	61.0	
Slight problems	9.4	5.1	6.8	12.4	18.0	27.5	29.4	
Moderate problems	1.0	0.9	1.4	1.8	2.8	6.4	9.6	
Severe problems	0.0	0.4	0.6	0.4	1.3	1.9	0.0	
Unable to	0.0	0.0	0.0	0.2	0.2	0.1	0.0	
**Self-care (%)**								<0.001
No problems	97.1	97.8	97.3	96.7	93.4	90.0	83.5	
Slight problems	2.2	1.7	2.1	2.7	5.6	8.7	11.2	
Moderate problems	0.8	0.4	0.4	0.2	0.6	0.9	5.4	
Severe problems	0.0	0.1	0.3	0.2	0.3	0.4	0.0	
Unable to	0.0	0.0	0.1	0.3	0.1	0.1	0.0	
**Activity (%)**								<0.001
No problems	91.4	92.9	91.0	89.4	85.0	74.2	79.4	
Slight problems	7.3	6.2	7.9	8.5	12.7	21.2	12.8	
Moderate problems	1.4	0.8	0.6	1.2	1.1	3.9	4.3	
Severe problems	0.0	0.0	0.5	0.4	0.9	0.4	3.5	
Unable to	0.0	0.1	0.1	0.4	0.4	0.2	0.0	
**Pain (%)**								<0.001
No problems	62.4	70.8	66.2	59.8	51.5	46.2	39.8	
Slight problems	30.6	25.3	28.3	33.1	40.1	41.4	37.2	
Moderate problems	4.0	3.6	4.1	5.8	6.6	10.7	23.0	
Severe problems	2.8	0.4	1.3	1.4	1.7	1.6	0.0	
Unable to	0.3	0.0	0.1	0.0	0.1	0.1	0.0	
**Anxiety (%)**								>0.1
No problems	85.1	85.8	85.5	84.4	84.3	83.0	86.1	
Slight problems	12.0	11.9	11.9	12.5	11.6	13.9	12.0	
Moderate problems	1.9	2.0	2.1	2.8	3.7	2.1	1.9	
Severe problems	1.0	0.4	0.5	0.3	0.4	0.7	0.0	
Unable to	0.0	0.0	0.1	0.0	0.0	0.3	0.0	

Table S7: Health-related quality of life in Bhutan by different socio-demographic characteristics, 2023

Variable	EQ index		EQ VAS	
	Mean (SD)	p-value [a]	Mean (SD)	p-value [a]
Gender		<0.001		<0.01
Female	0.95 (0.11)		76.69 (17.51)	
Male	0.95 (0.09)		77.68 (17.32)	
Age, years		<0.001		<0.001
15-19	0.96 (0.08)		78.53 (16.45)	
20-29	0.97 (0.07)		79.68 (16.43)	
30-39	0.96 (0.09)		78.70 (17.24)	
40-49	0.95 (0.10)		76.80 (17.41)	
50-59	0.94 (0.12)		74.36 (17.64)	
60-69	0.91 (0.13)		72.94 (18.25)	
≥70	0.88 (0.17)		67.73 (17.90)	
Education level		<0.001		<0.001
No formal education	0.93 (0.12)		74.87 (17.89)	
Primary or less	0.95 (0.10)		76.41 (17.54)	
Secondary or equivalent	0.96 (0.08)		78.89 (16.85)	
Tertiary	0.97 (0.07)		80.87 (16.04)	
Marital status		<0.001		<0.001
Never married	0.95 (0.11)		78.10 (17.18)	
Married	0.95 (0.10)		77.31 (17.37)	
Others*	0.93 (0.14)		74.06 (17.94)	
Working status		<0.001		<0.001
Working	0.95 (0.09)		77.91 (17.27)	
Not working	0.94 (0.12)		76.02 (17.59)	
Body mass index		>0.1		>0.1
Underweight	0.96 (0.09)		77.08 (17.93)	
Normal weight	0.95 (0.11)		76.68 (17.60)	
Overweight	0.95 (0.10)		77.52 (17.05)	
Obese	0.95 (0.10)		76.92 (17.81)	
Blood pressure		>0.05		>0.1
Poor	0.95 (0.09)		77.15 (17.10)	
Intermediate	0.95 (0.11)		77.21 (17.27)	
Ideal	0.95 (0.10)		76.85 (17.87)	
Fruit consumption		>0.1		>0.1
Poor	0.95 (0.10)		76.90 (17.30)	
Intermediate	0.95 (0.10)		77.23 (17.45)	
Ideal	0.95 (0.11)		76.95 (17.54)	
Vegetable consumption		>0.1		>0.1
Poor	0.94 (0.10)		76.67 (17.98)	
Intermediate	0.95 (0.13)		76.78 (17.53)	
Ideal	0.95 (0.10)		77.08 (17.43)	
Expenditure quintile		<0.01		<0.01
Quintile 1 (poorest)	0.95 (0.10)		77.29 (17.08)	
Quintile 2	0.95 (0.12)		77.14 (17.49)	
Quintile 3	0.95 (0.10)		77.72 (17.41)	
Quintile 4	0.95 (0.10)		77.43 (17.26)	
Quintile 5 (richest)	0.94 (0.11)		75.77 (17.99)	
Place of residence		<0.001		<0.001
Urban	0.96 (0.08)		79.19 (16.91)	
Rural	0.94 (0.11)		75.82 (17.63)	
Region of residence		<0.001		<0.01
East	0.95 (0.10)		76.11 (17.64)	
West	0.96 (0.09)		77.25 (17.30)	
Central	0.94 (0.12)		77.58 (17.49)	
**Total**	**0.95 (0.10)**	**NA**	**77.06 (17.44)**	**NA**

[a] Note: This is the p-value of the F-statistic. P-value is significant when ≤ 0.05.

*Others refers to divorced/widowed/separated

Table S8: Mean health-related quality of life using Indian and Thai value set, by district

District	Indian_set_value	Thai_set_value	mean_differences	p_value
Bumthang	0.948	0.874	-0.074 (-0.076, -0.072)	<0.001
Chhukha	0.952	0.876	-0.076 (-0.078, -0.074)	<0.001
Dagana	0.929	0.856	-0.073 (-0.076, -0.070)	<0.001
Gasa	0.947	0.875	-0.072 (-0.074, -0.070)	<0.001
Haa	0.966	0.891	-0.075 (-0.077, -0.073)	<0.001
Lhuentse	0.931	0.865	-0.066 (-0.070, -0.062)	<0.001
Monggar	0.947	0.869	-0.078 (-0.080, -0.076)	<0.001
Paro	0.946	0.873	-0.073 (-0.077, -0.070)	<0.001
Pema Gatshel	0.934	0.864	-0.070 (-0.074, -0.067)	<0.001
Punakha	0.949	0.870	-0.079 (-0.081, -0.078)	<0.001
Samdrup Jongkhar	0.979	0.900	-0.079 (-0.080, -0.078)	<0.001
Samtse	0.951	0.877	-0.075 (-0.076, -0.073)	<0.001
Sarpang	0.966	0.891	-0.076 (-0.077, -0.074)	<0.001
Thimphu	0.963	0.886	-0.077 (-0.078, -0.076)	<0.001
Trashigang	0.941	0.869	-0.072 (-0.074, -0.070)	<0.001
Trashi Yangtse	0.944	0.868	-0.075 (-0.078, -0.073)	<0.001
Trongsa	0.936	0.859	-0.076 (-0.078, -0.075)	<0.001
Tsirang	0.950	0.877	-0.074 (-0.077, -0.071)	<0.001
Wangdue Phodrang	0.881	0.823	-0.058 (-0.063, -0.053)	<0.001
Zhemgang	0.961	0.885	-0.076 (-0.079, -0.073)	<0.001
National	0.950	0.875	-0.074 (-0.075, -0.074)	<0.001

Note: The mean difference was calculated as the survey-weighted mean of the individual-level difference between the two indices, defined as EQindex_Thai − EQindex, for each district (and nationally). The p-values were obtained by testing the null hypothesis that this weighted mean difference equals zero, using the ratio of the estimated mean difference to its survey-weighted standard error (t-test), with p-values reported to three decimal places.

Table S9: Regression analyses on health-related quality of life scores using Thai value set

	EQ-5D score (Tobit regression)		
**Predictors**	**Estimates**	**95% CI**	**p**
**Age (years)**			
15-19	Ref.		
20-29	-0.00	-0.01 – 0.01	0.83
30-39	-0.01	-0.01 – 0.00	0.19
40-49	-0.01	-0.02 – -0.00	<0.01
50-59	-0.02	-0.03 – -0.01	<0.01
60-69	-0.04	-0.05 – -0.03	<0.01
≥70	-0.06	-0.08 – -0.04	<0.01
**Education**			
No formal education	Ref.		
Primary or less	0.00	0.00 – 0.01	0.03
Secondary or equivalent	0.01	0.00 – 0.01	<0.01
Tertiary	0.02	0.01 – 0.02	<0.01
**Marital status**			
Never married	Ref.		
Married	0.01	0.01 – 0.02	<0.01
Others*	-0.00	-0.01 – 0.01	0.80
**Working status**			
Working	Ref.		
Not working	-0.01	-0.01 – -0.00	<0.01
**Body mass index**			
Underweight	Ref.		
Normal weight	-0.01	-0.01 – 0.00	0.23
Overweight	-0.00	-0.01 – 0.00	0.36
Obese	-0.00	-0.01 – 0.01	0.55
**Blood pressure**			
Poor	Ref.		
Intermediate	-0.00	-0.01 – 0.00	0.20
Ideal	-0.00	-0.01 – 0.00	0.08
**Fruit intake**			
Poor	Ref.		
Intermediate	0.01	0.00 – 0.01	<0.01
Ideal	0.01	0.00 – 0.01	<0.01
**Expenditure quintile**			
Quintile 1 (poorest)	Ref.		
Quintile 2	-0.00	-0.01 – 0.00	0.31
Quintile 3	-0.00	-0.01 – 0.00	0.05
Quintile 4	-0.01	-0.01 – -0.00	0.03
Quintile 5 (richest)	-0.01	-0.02 – -0.01	<0.01
**Area of residence**			
Urban	Ref.		
Rural	-0.01	-0.01 – -0.01	<0.01
R2 Nagelkerke	-0.006		

Table S10: inequalities in EQ-5D-5L health domain across different sociodemographic variables, 2023

Equity strata	SII (95% confidenc interval)		
	Both	Male	Female
**Age (15-19 vs ≥70)**			
Mobility	23.99 (20.89–27.08)	17.21 (12.72–21.70)	28.98 (24.94–33.03)
Self-care	6.76 (4.85–8.67)	6.33 (3.37–9.29)	7.20 (4.86–9.54)
Activity	13.73 (11.07–16.38)	10.00 (5.87–14.12)	16.62 (13.21–20.04)
Pain	25.13 (21.22–29.04)	23.07 (17.39–28.74)	27.30 (22.26–32.35)
Anxiety	1.93 (-0.79–4.64)	-0.30 (-4.18–3.58)	3.88 (0.32–7.44)
**Education (No vs High)**			
Mobility	-21.23 (-24.25– -18.20)	-19.61 (-24.03– -15.19)	-22.38 (-26.21– -18.55)
Self-care	-4.93 (-6.81– -3.05)	-4.70 (-7.36– -2.04)	-5.02 (-7.46– -2.58)
Activity	-14.84 (-17.56– -12.12)	-14.10 (-18.39– -9.81)	-15.16 (-18.58– -11.75)
Pain	-20.56 (-24.00– -17.18)	-20.70 (-26.09– -15.32)	-19.78 (-24.19– -15.37)
Anxiety	-5.64 (-8.53– -2.75)	-5.38 (-9.71– -1.05)	-5.22 (-9.13– -1.32)
**Qunitle (Q1 vs Q5)**			
Mobility	0.82 (-2.23–3.87)	-1.01 (-5.58–3.56)	1.97 (-1.92–5.86)
Self-care	-0.88 (-2.76–1.00)	-1.44 (-4.34–1.45)	-0.53 (-2.95–1.89)
Activity	0.38 (-2.57–3.33)	1.54 (-2.80–5.89)	-0.35 (-4.06–3.36)
Pain	4.98 (0.52–9.45)	3.04 (-3.47–9.55)	6.19 (0.79–11.60)
Anxiety	1.30 (-1.80–4.39)	-0.03 (-4.31–4.24)	2.13 (-2.01–6.26)

SII, slope index of inequalities

Table S11: Inequalities in EQ-5D-5L index by area of residence, 2023

Subnational	Mean (EQ-5D-5L index)			SII (95% CI)	CIX (95% CI)
	Total	Urban	Rural		
Bumthang	0.948	0.958	0.941	-3.49 (-7.17–0.19)	-0.004 (-0.009–0.000)
Chhukha	0.952	0.954	0.950	-0.77 (-3.3–1.75)	-0.001 (-0.006–0.004)
Dagana	0.929	0.973	0.922	-10.32 (-14.36–-6.27)	-0.006 (-0.009–-0.004)
Gasa	0.947	0.963	0.940	-4.72 (-7.59–-1.86)	-0.005 (-0.010–-0.001)
Haa	0.966	0.976	0.963	-2.59 (-5.7–0.52)	-0.003 (-0.005–0.000)
Lhuentse	0.931	0.936	0.930	-1.09 (-9.59–7.41)	-0.001 (-0.005–0.004)
Monggar	0.947	0.976	0.937	-7.85 (-11.15–-4.55)	-0.008 (-0.012–-0.003)
Paro	0.946	0.972	0.938	-6.79 (-10.36–-3.23)	-0.007 (-0.011–-0.002)
Pema Gatshel	0.934	0.935	0.933	-0.41 (-6.54–5.71)	-0.000 (-0.006–0.006)
Punakha	0.949	0.961	0.946	-2.9 (-6.29–0.49)	-0.002 (-0.005–0.001)
Samdrup Jongkhar	0.979	0.982	0.977	-0.93 (-2.44–0.58)	-0.001 (-0.003–0.001)
Samtse	0.951	0.969	0.948	-4.26 (-6.44–-2.09)	-0.003 (-0.005–-0.001)
Sarpang	0.966	0.972	0.964	-1.61 (-3.96–0.74)	-0.002 (-0.005–0.002)
Thimphu	0.963	0.961	0.972	2.2 (0.17–4.23)	0.002 (-0.000–0.004)
Trashigang	0.941	0.968	0.937	-6.43 (-9.23–-3.63)	-0.004 (-0.006–-0.002)
Trashi Yangtse	0.944	0.951	0.942	-1.8 (-7.67–4.08)	-0.001 (-0.005–0.002)
Trongsa	0.936	0.943	0.934	-1.83 (-5.9–2.24)	-0.002 (-0.005–0.002)
Tsirang	0.950	0.952	0.950	-0.49 (-7.39–6.42)	-0.000 (-0.004–0.004)
Wangdue Phodrang	0.881	0.920	0.863	-11.53 (-17.44–-5.62)	-0.014 (-0.023–-0.005)
Zhemgang	0.961	0.984	0.952	-6.18 (-10.27–-2.08)	-0.007 (-0.010–-0.003)
National	0.950	0.961	0.943	-3.39 (-4.69–-2)	-0.004 (-0.006–-0.003)

SII, slope index of inequalities; CI, confidence interval

Table S12: Inequalities in EQ-5D-5L index by educational level, 2023

	Mean (EQ-5D-5L index)					
Subnational	No education	Primary or below	Secondary or equivalent	Tertiary	SII (95% CI)	CIX (95% CI)
Bumthang	0.926	0.957	0.962	0.965	5.91 (2.3–9.52)	0.009 (0.004–0.015)
Chhukha	0.941	0.945	0.960	0.964	3.41 (0.66–6.17)	0.005 (0.001–0.009)
Dagana	0.910	0.933	0.952	1.000	8.22 (3.46–12.98)	0.013 (0.005–0.021)
Gasa	0.924	0.972	0.967	0.958	6.99 (4.05–9.93)	0.011 (0.004–0.017)
Haa	0.953	0.981	0.971	0.980	3.36 (0.24–6.48)	0.005 (0.002–0.009)
Lhuentse	0.915	0.935	0.959	0.989	8.64 (4.41–12.86)	0.012 (0.006–0.017)
Monggar	0.932	0.942	0.969	0.986	5.9 (2.52–9.29)	0.009 (0.004–0.014)
Paro	0.924	0.936	0.957	0.981	6.81 (2.23–11.38)	0.010 (0.004–0.017)
Pema Gatshel	0.929	0.923	0.947	0.939	2.56 (-0.78–5.9)	0.004 (-0.001–0.009)
Punakha	0.936	0.948	0.963	0.968	4.95 (2.02–7.89)	0.007 (0.003–0.012)
Samdrup Jongkhar	0.974	0.988	0.981	0.994	1.56 (0.11–3.02)	0.002 (0.000–0.004)
Samtse	0.945	0.947	0.962	0.970	2.96 (0.75–5.17)	0.004 (0.000–0.008)
Sarpang	0.957	0.961	0.977	0.988	3.78 (1.74–5.81)	0.006 (0.003–0.009)
Thimphu	0.955	0.963	0.964	0.971	1.81 (0.43–3.18)	0.003 (0.001–0.005)
Trashigang	0.928	0.940	0.970	0.972	5.99 (3.19–8.8)	0.009 (0.005–0.013)
Trashi Yangtse	0.941	0.940	0.948	0.959	1.52 (-2.19–5.24)	0.002 (-0.002–0.007)
Trongsa	0.912	0.957	0.954	0.968	7.96 (4.38–11.54)	0.012 (0.005–0.019)
Tsirang	0.940	0.952	0.958	0.980	3.61 (-0.84–8.05)	0.006 (-0.002–0.013)
Wangdue Phodrang	0.844	0.901	0.925	0.914	14.05 (8.22–19.89)	0.022 (0.014–0.030)
Zhemgang	0.958	0.973	0.957	0.983	0.5 (-3.77–4.76)	0.001 (-0.005–0.007)
National	0.935	0.948	0.962	0.972	5.14 (4.21–6.07)	0.008 (0.006–0.009)

SII, slope index of inequalities; CI, confidence interval; CIX, concentration index

Table S13: Inequalities in EQ-5D-5L index by expenditure quintile, 2023

Subnational	Mean (EQ-5D-5L index)					SII (95% CI)	CIX (95% CI)
	Q1	Q2	Q3	Q4	Q5		
Bumthang	0.970	0.970	0.964	0.947	0.924	-6.62 (-9.95–-3.28)	-0.011 (-0.018–-0.004)
Chhukha	0.954	0.947	0.954	0.966	0.942	-0.49 (-2.79–1.81)	-0.001 (-0.005–0.003)
Dagana	0.942	0.942	0.891	0.914	0.929	-4.22 (-9.07–0.63)	-0.007 (-0.015–0.001)
Gasa	0.943	0.958	0.944	0.951	0.942	-0.3 (-3.36–2.76)	-0.000 (-0.006–0.005)
Haa	0.976	0.978	0.966	0.937	0.890	-5.26 (-8.88–-1.64)	-0.009 (-0.017–-0.000)
Lhuentse	0.931	0.906	0.926	0.949	0.943	2.96 (-1.3–7.22)	0.005 (-0.004–0.014)
Monggar	0.950	0.940	0.954	0.947	0.940	-0.53 (-3.6–2.55)	-0.001 (-0.005–0.004)
Paro	0.959	0.948	0.955	0.937	0.945	-1.12 (-4.86–2.62)	-0.002 (-0.006–0.003)
Pema Gatshel	0.935	0.926	0.924	0.956	0.941	1.66 (-1.61–4.93)	0.003 (-0.003–0.009)
Punakha	0.947	0.953	0.958	0.951	0.915	-1.05 (-4.85–2.75)	-0.002 (-0.008–0.005)
Samdrup Jongkhar	0.973	0.983	0.985	0.980	0.975	0.41 (-1.19–2)	0.001 (-0.002–0.004)
Samtse	0.947	0.953	0.957	0.946	0.967	1.21 (-0.93–3.35)	0.002 (-0.001–0.005)
Sarpang	0.961	0.956	0.974	0.971	0.967	1.42 (-0.52–3.36)	0.002 (-0.001–0.005)
Thimphu	0.974	0.970	0.963	0.961	0.959	-1.44 (-2.56–-0.31)	-0.002 (-0.004–-0.000)
Trashigang	0.951	0.931	0.954	0.948	0.922	-1.51 (-4.75–1.72)	-0.003 (-0.009–0.004)
Trashi Yangtse	0.950	0.954	0.921	0.965	0.912	-2.94 (-6.54–0.65)	-0.005 (-0.010–0.000)
Trongsa	0.935	0.950	0.927	0.961	0.899	-2.29 (-6.26–1.69)	-0.004 (-0.011–0.003)
Tsirang	0.963	0.973	0.961	0.934	0.906	-6.96 (-12.17–-1.75)	-0.012 (-0.022–-0.002)
Wangdue Phodrang	0.863	0.873	0.861	0.879	0.909	5.94 (0.28–11.61)	0.011 (-0.001–0.023)
Zhemgang	0.962	0.964	0.964	0.938	0.966	-0.62 (-4.93–3.68)	-0.001 (-0.007–0.005)
National	0.950	0.949	0.952	0.952	0.944	-0.91 (-1.83–0.02)	-0.001 (-0.002–0.000)

SII, slope index of inequalities; CI, confidence interval; CIX, concentration index

Table S14: Inequalities in EQ VAS score by area of residence, 2023

Districts	Mean VAS score			SII (95% CI)	CIX (95% CI)
	Total	Urban	Rural		
Bumthang	82.88	83.51	82.43	-2.15 (-8.27–3.97)	-0.003 (-0.013–0.006)
Chhukha	78.38	81.52	74.56	-14.02 (-18.23–-9.81)	-0.022 (-0.032–-0.012)
Dagana	78.97	80.7	78.7	-3.97 (-13.06–5.11)	-0.003 (-0.007–0.001)
Gasa	78.43	81.01	77.19	-7.42 (-14.88–0.04)	-0.011 (-0.019–-0.003)
Haa	83.03	84.47	82.55	-3.73 (-11.57–4.11)	-0.004 (-0.012–0.003)
Lhuentse	72.24	75.08	71.84	-6.51 (-17.93–4.91)	-0.005 (-0.010–0.001)
Monggar	83.49	86.68	82.42	-8.53 (-14.89–-2.16)	-0.010 (-0.024–0.005)
Paro	73.63	74.38	73.4	-1.96 (-9.19–5.28)	-0.002 (-0.014–0.009)
Pema Gatshel	71.55	72.11	71.33	-1.56 (-9.07–5.95)	-0.002 (-0.010–0.006)
Punakha	75.99	82.95	74.47	-16.87 (-24.18–-9.56)	-0.016 (-0.023–-0.010)
Samdrup Jongkhar	76.57	80.76	73.88	-13.84 (-18.47–-9.2)	-0.021 (-0.028–-0.014)
Samtse	76.33	79.08	75.76	-6.67 (-11.57—1.76)	-0.006 (-0.011–-0.002)
Sarpang	79	80.2	78.61	-3.25 (-8.22–1.72)	-0.004 (-0.013–0.005)
Thimphu	78.21	77.9	79.6	3.45 (-0.68–7.57)	0.003 (-0.001–0.008)
Trashigang	74.63	80.27	73.62	-13.51 (-21.06–-5.95)	-0.011 (-0.018–-0.005)
Trashi Yangtse	73.07	74.75	72.71	-4.11 (-14.13–5.91)	-0.004 (-0.013–0.004)
Trongsa	80.41	81.83	80.04	-3.57 (-9.61–2.46)	-0.004 (-0.009–0.002)
Tsirang	78.93	81.95	78.54	-6.77 (-16.46–2.93)	-0.004 (-0.008–-0.001)
Wangdue Phodrang	72.62	78.06	70.13	-15.82 (-23.38–-8.25)	-0.024 (-0.038–-0.009)
Zhemgang	72.8	79.42	70.23	-18.3 (-28.31—8.29)	-0.026 (-0.037–-0.014)
**National**	**77.06**	**79.19**	**75.82**	**-8.89 (-11.31—6.46)**	**-0.010 (-0.013–-0.007)**

VAS, Visual Analogue Scale; SII, slope index of inequalities; CI, confidence interval; CIX, concentration index

Table S15: Inequalities in EQ VAS score by educational level, 2023

Districts	Mean VAS score				SII (95% CI)	CIX (95% CI)
	No education	Primary or below	Secondary or equivalent	Tertiary		
Bumthang	79.37	83.16	85.43	85.78	10.04 (4.63–15.45)	0.018 (0.009–0.027)
Chhukha	75.03	77.51	80.03	83.76	10.5 (6.48–14.52)	0.019 (0.011–0.027)
Dagana	76.99	78.27	81.39	91.21	9.68 (3.15–16.21)	0.017 (0.007–0.028)
Gasa	74.22	85.82	80.15	84.54	12.82 (5.55–20.09)	0.023 (0.010–0.037)
Haa	81.36	86.03	84.38	79.21	2.7 (-4.36–9.75)	0.005 (-0.008–0.018)
Lhuentse	70.74	69.43	75.77	81.11	9.61 (1.97–17.26)	0.017 (0.003–0.031)
Monggar	81.6	83.01	85.47	94.91	7.97 (2.57–13.37)	0.014 (0.002–0.026)
Paro	72.16	70.5	74.88	76.65	6.08 (-0.25–12.41)	0.012 (-0.002–0.026)
Pema Gatshel	70.47	73.1	72.05	74.22	3.73 (-2.91–10.38)	0.007 (-0.008–0.023)
Punakha	72.71	74.6	80.23	78.9	12.31 (5.85–18.77)	0.023 (0.010–0.036)
Samdrup Jongkhar	73.63	80.24	78.42	82.24	9.6 (5.31–13.89)	0.018 (0.009–0.027)
Samtse	75.6	75.37	77.33	82.87	4.13 (0.23–8.03)	0.008 (0.001–0.015)
Sarpang	78.76	77.78	79.81	79.24	1.45 (-3.36–6.26)	0.003 (-0.008–0.013)
Thimphu	76.13	76.45	79.11	79.37	4.77 (1.56–7.97)	0.009 (0.002–0.015)
Trashigang	73.5	71.8	79.18	84.39	7.79 (2.82–12.76)	0.015 (0.006–0.024)
Trashi Yangtse	69.23	78.39	75.15	81.8	13.06 (5.47–20.64)	0.025 (0.011–0.039)
Trongsa	77.41	80.92	82.94	86.34	11.17 (5.98–16.36)	0.019 (0.013–0.026)
Tsirang	77.6	80.39	79.63	80.17	3.65 (-2.98–10.27)	0.007 (-0.004–0.017)
Wangdue Phodrang	68.48	76.23	76.67	79.2	15.19 (8.85–21.53)	0.029 (0.015–0.044)
Zhemgang	70.47	68.89	75.53	88.14	11.89 (3.9–19.88)	0.024 (0.009–0.039)
**National**	**74.87**	**76.41**	**78.89**	**80.87**	**8.88 (7.29–10.47)**	**0.015 (0.012–0.017)**

VAS, Visual Analogue Scale; SII, slope index of inequalities; CI, confidence interval; CIX, concentration index

Table S16: Inequalities in EQ VAS score by expenditure quintile, 2023

Districts	Mean VAS score					SII (95% CI)	CIX (95% CI)
	Q1	Q2	Q3	Q4	Q5		
Bumthang	77.47	87.1	82.81	81.96	82.77	-0.91 (-6.82–4.99)	-0.002 (-0.016–0.012)
Chhukha	78.29	78.28	81.29	79.68	74.9	-4.04 (-7.81–-0.28)	-0.008 (-0.018–0.002)
Dagana	78.76	77.71	78.92	81.52	79.27	2.06 (-4.31–8.42)	0.004 (-0.006–0.014)
Gasa	79.09	76.26	77.15	82.25	78.71	2.21 (-5.06–9.47)	0.004 (-0.013–0.021)
Haa	84.25	84.65	82.97	76.64	86.44	-6.14 (-12.61–0.33)	-0.011 (-0.021–-0.002)
Lhuentse	77.61	71.66	75.43	73.09	64.64	-14.59 (-21.68–-7.5)	-0.032 (-0.048–-0.016)
Monggar	85.72	83.94	81.27	80.82	81.05	-7.17 (-12.52–-1.81)	-0.013 (-0.026–-0.001)
Paro	76.57	75.7	73.51	73.28	72.65	-4.2 (-9.89–1.49)	-0.009 (-0.020–0.002)
Pema Gatshel	70.79	70.47	71.88	74.71	71.02	2.94 (-3.27–9.14)	0.007 (-0.005–0.018)
Punakha	75.79	77.48	78.32	75.41	66.75	-4.26 (-10.43–1.91)	-0.009 (-0.022–0.005)
Samdrup Jongkhar	75.9	76.4	77.77	77.03	75.74	0.68 (-3.53–4.9)	0.001 (-0.007–0.010)
Samtse	77.4	74.45	76.47	74.75	79.36	-1.65 (-5.49–2.19)	-0.003 (-0.012–0.005)
Sarpang	79.57	79.26	79.68	77.06	80.31	-1.12 (-5.61–3.38)	-0.002 (-0.012–0.007)
Thimphu	80.48	79.04	78.2	79.01	76.81	-3.33 (-6.54–-0.12)	-0.007 (-0.014–0.000)
Trashigang	77.22	74.32	74.22	75.25	72.91	-2.6 (-7.66–2.45)	-0.006 (-0.016–0.005)
Trashi Yangtse	69.59	74.68	73.42	80.84	69.41	6.39 (-0.87–13.64)	0.014 (-0.003–0.031)
Trongsa	81.22	80.37	80.41	81.52	78.08	-2.09 (-7.12–2.95)	-0.004 (-0.012–0.004)
Tsirang	79.18	81.74	80.62	77.73	74.17	-5.9 (-12.39–0.59)	-0.012 (-0.032–0.008)
Wangdue Phodrang	70.21	73.26	68.83	73.23	75.32	6.06 (-0.29–12.42)	0.013 (-0.002–0.028)
Zhemgang	74.32	72.69	75.16	64.96	69.32	-5.78 (-13.94–2.38)	-0.012 (-0.030–0.006)
**National**	**77.91**	**77.31**	**77.48**	**77.19**	**75.51**	**-1.79 (-3.37–-0.2)**	-0.005 (-0.008–-0.002)

VAS, Visual Analogue Scale; SII, slope index of inequalities; CI, confidence interval; CIX, concentration index

Figure S1: National and subnational level disparities in EQ-5D score

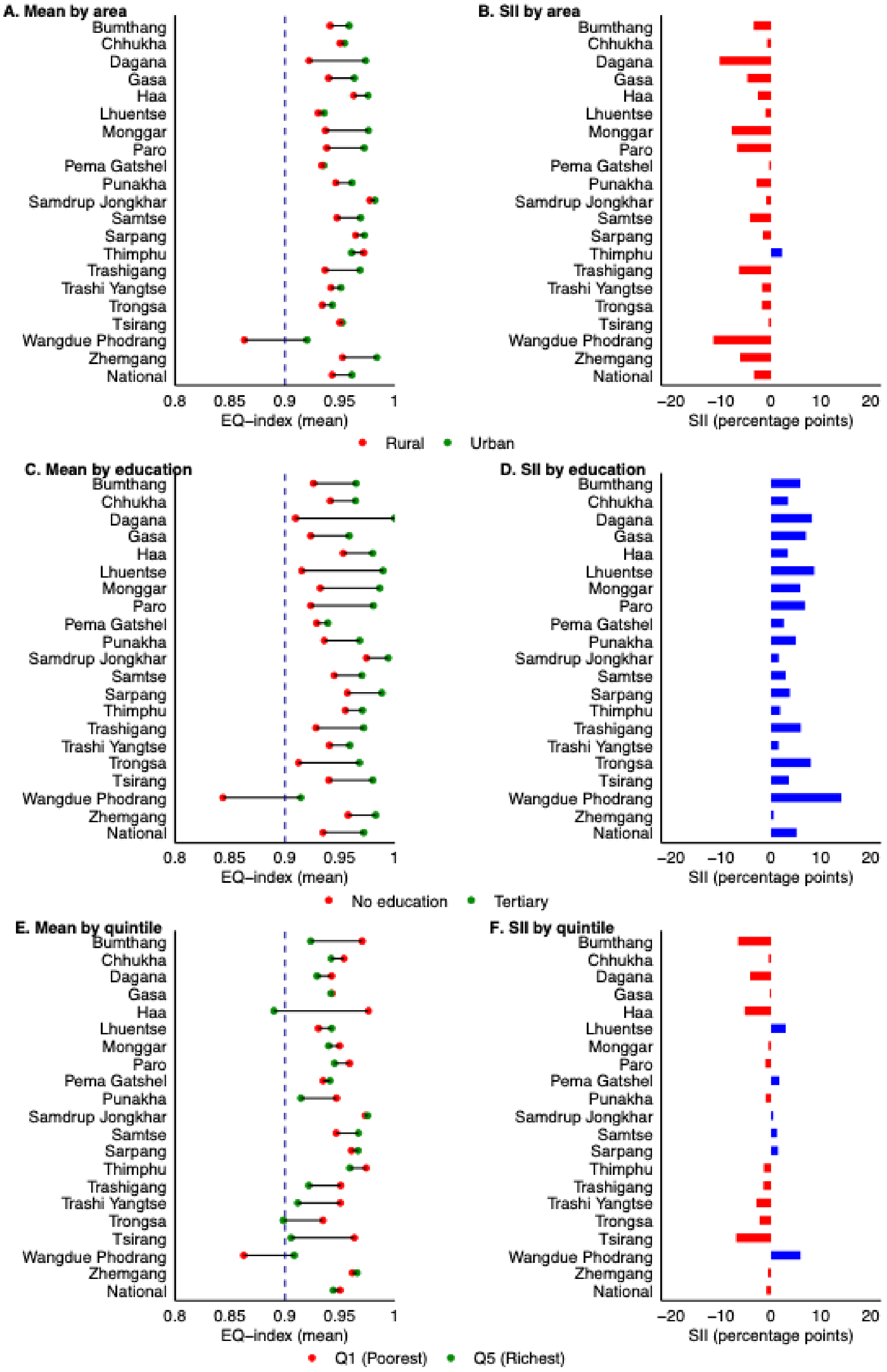

SII, slope index of inequalities

Figure S2: National and subnational level disparities in EQ-VAS score

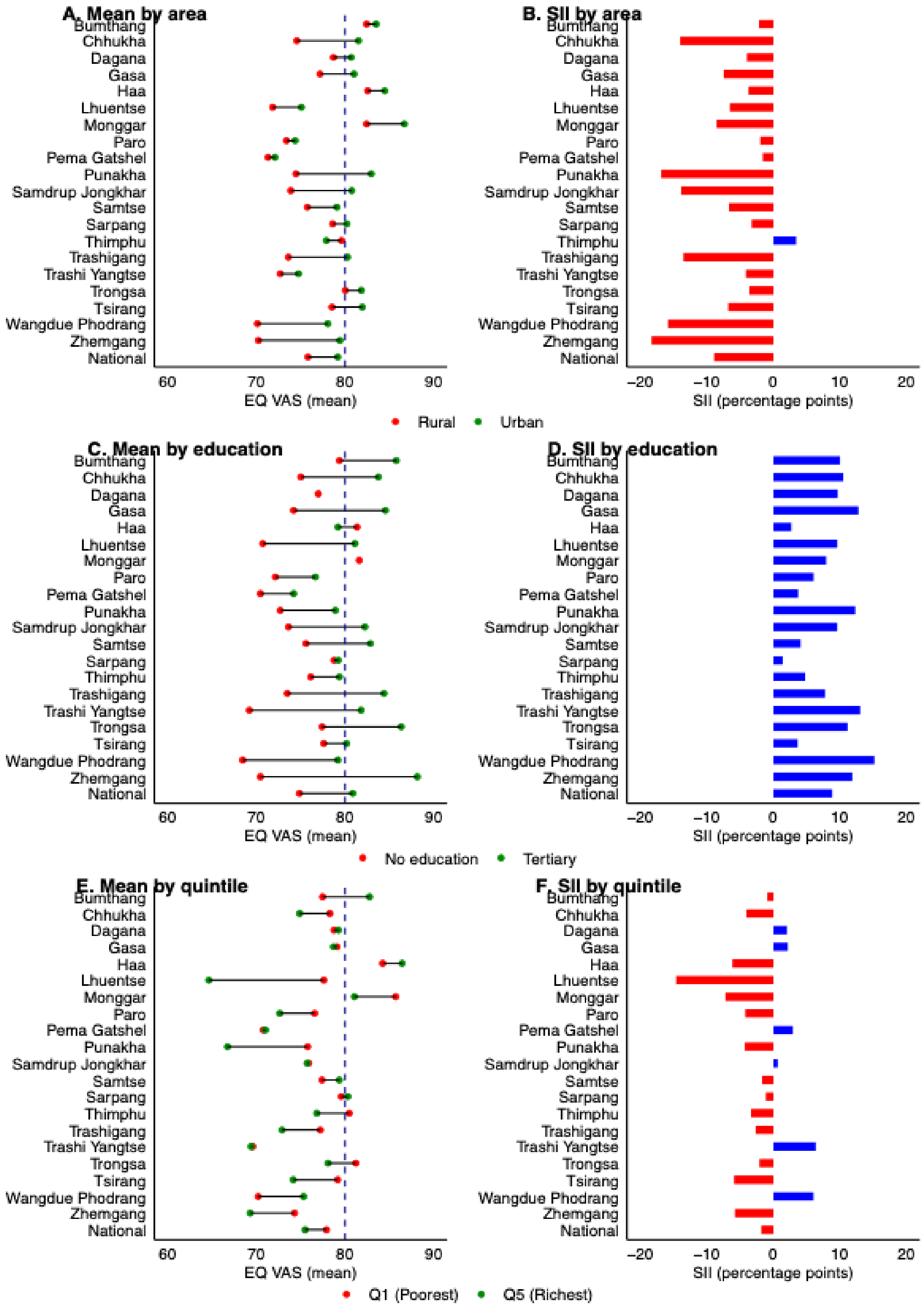

VAS, Visual Analogue Scale; SII, slope index of inequalities

Figure S3: Relative index of inequalities in EQ-5D score

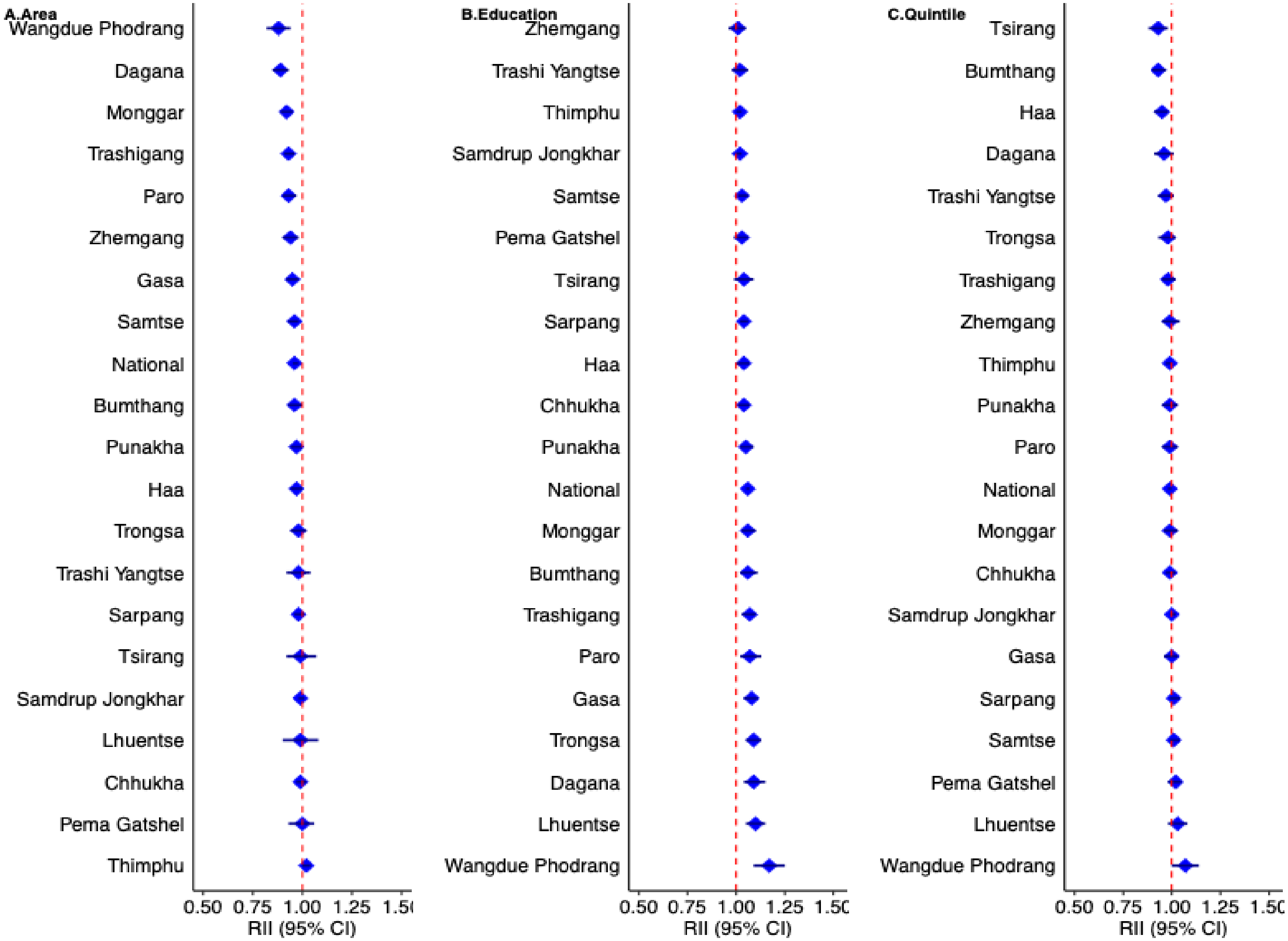

RII, slope index of inequalities; CI, confidence interval

Figure S4: Relative index of inequalities in EQ-VAS

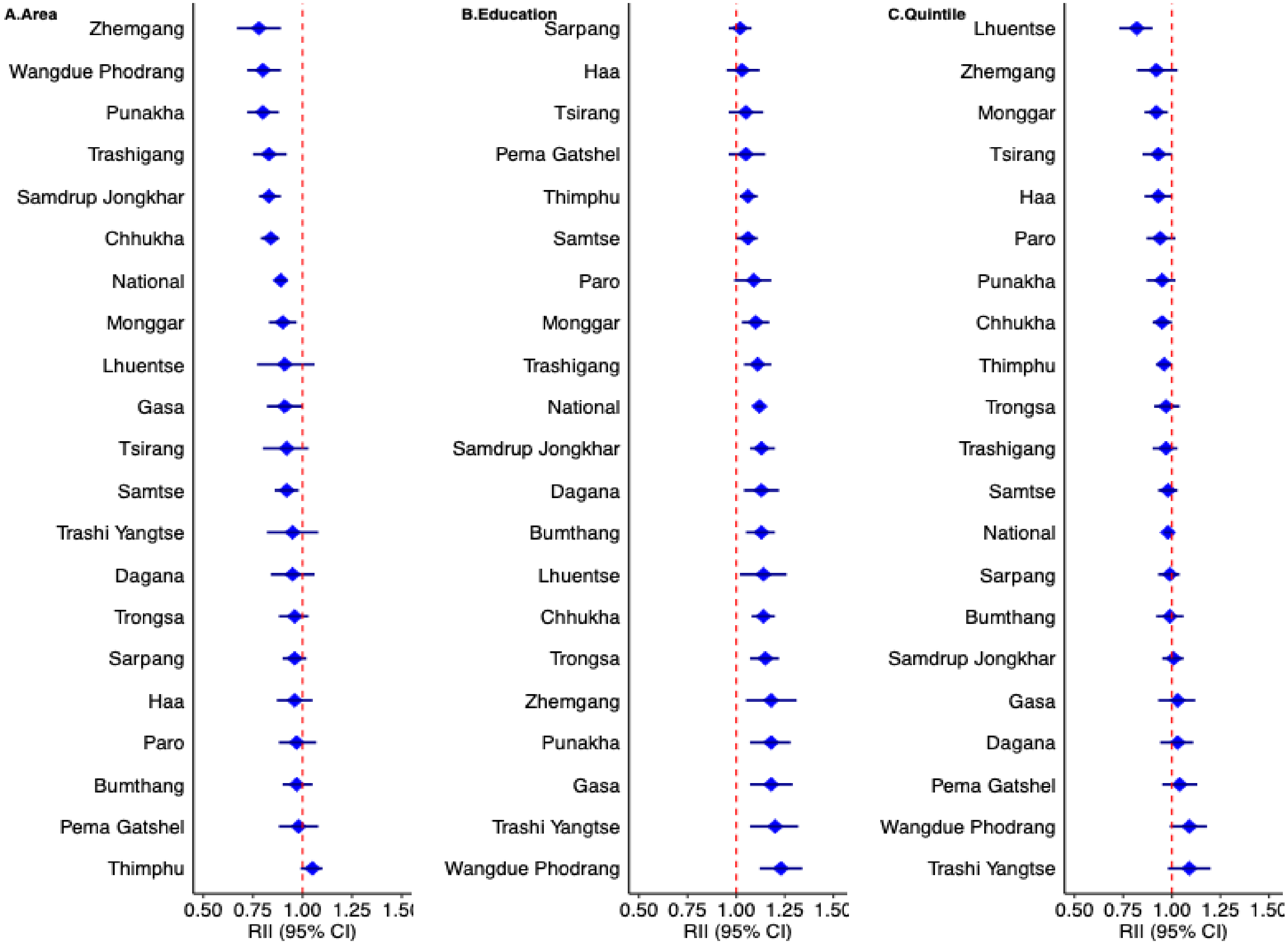

VAS, Visual Analogue Scale; RII, slope index of inequalities; CI, confidence interval
